# In Situ Biofilm Affinity-Based Protein Profiling Identifies the Streptococcal Hydrolase GbpB as the Target of a Carolacton-Inspired Chemical Probe

**DOI:** 10.1021/jacs.4c06658

**Published:** 2024-08-12

**Authors:** Amber M. Scharnow, Amy E. Solinski, Sebastian Rowe, Ines Drechsel, Hua Zhang, Elana Shaw, Julia E. Page, Hui Wu, Stephan A. Sieber, William M. Wuest

**Affiliations:** † Department of Chemistry, Emory University, Atlanta, Georgia 30322, United States; ‡ Department of Chemical Biology, Harvard University, Cambridge, Massachusetts 02138, United States; § Department of Chemistry, Center for Functional Protein Assemblies, Technical University of Munich, Garching D-85747, Germany; ∥ Departments of Pediatric Dentistry, Microbiology, Schools of Dentistry and Medicine, University of Alabama at Birmingham, Birmingham 35294, Alabama, United States; ⊥ Department of Microbiology, Blavatnik Institute, Harvard Medical School, Boston, Massachusetts 02115, United States

## Abstract

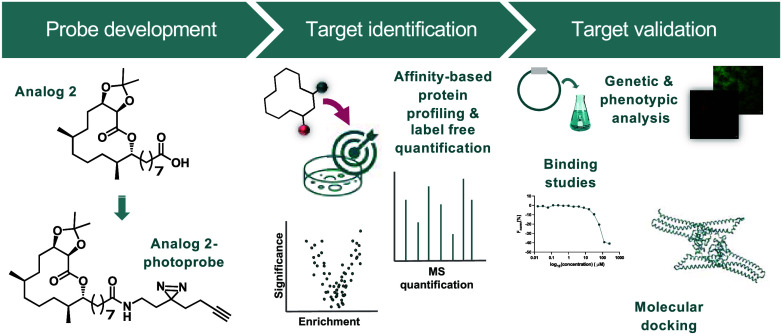

Natural products are important precursors for antibiotic drug design. These chemical scaffolds serve as synthetic inspiration for chemists who leverage their structures to develop novel antibacterials and chemical probes. We have previously studied carolacton, a natural product macrolactone from*Sorangium cellulosum*, and discovered a simplified derivative, **A2**, that maintained apparent biofilm inhibitory activity, although the biological target was unknown. Herein, we utilize affinity-based protein profiling (AfBPP) *in situ* during biofilm formation to identify the protein target using a photoexcitable cross-linking derivative of **A2**. From these studies, we identified glucan binding protein B (GbpB), a peptidoglycan hydrolase, as the primary target of **A2**. Further characterization of the interaction between **A2** and GbpB, as well as PcsB, a closely related homologue from the more pathogenic *S. pneumoniae*, revealed binding to the catalytic CHAP (cysteine, histidine, aminopeptidase) domain. To the best of our knowledge, this is the first report of a small-molecule binder of a conserved and essential bacterial CHAP hydrolase, revealing its potential as an antibiotic target. This work also highlights **A2** as a useful tool compound for streptococci and as an initial scaffold for the design of more potent CHAP binders.

## Introduction

Antibiotic development has been impeded by an increase in multidrug resistant organisms and a decline in investment. Natural products are often considered “privileged scaffolds”, as they have been evolved to impart specific binding with therapeutically relevant targets, evidenced by the fact that 65% of approved antibacterials are derived from natural products.^1^ Unfortunately, they are limited in their practical use due to their poor therapeutic indices, bioavailability, and synthetic accessibility.^2,3^ To overcome these challenges, many have been motivated to develop synthetic strategies to leverage their advantages in biological settings through the construction of natural product-inspired libraries. Examples include biology-oriented synthesis (BOS),^4,5^ diverted total synthesis (DTS),^6,7^ diversity-oriented synthesis (DOS),^8,9^ complexity to diversity (CtD),^10,11^ and natural product simplification (NPS).^12−15^ Such chemical platforms have enabled the design of chemical probes, the identification of unexplored targets, and the development of antibiotics that circumvent resistance.^3,16−23^

**Figure 1:**
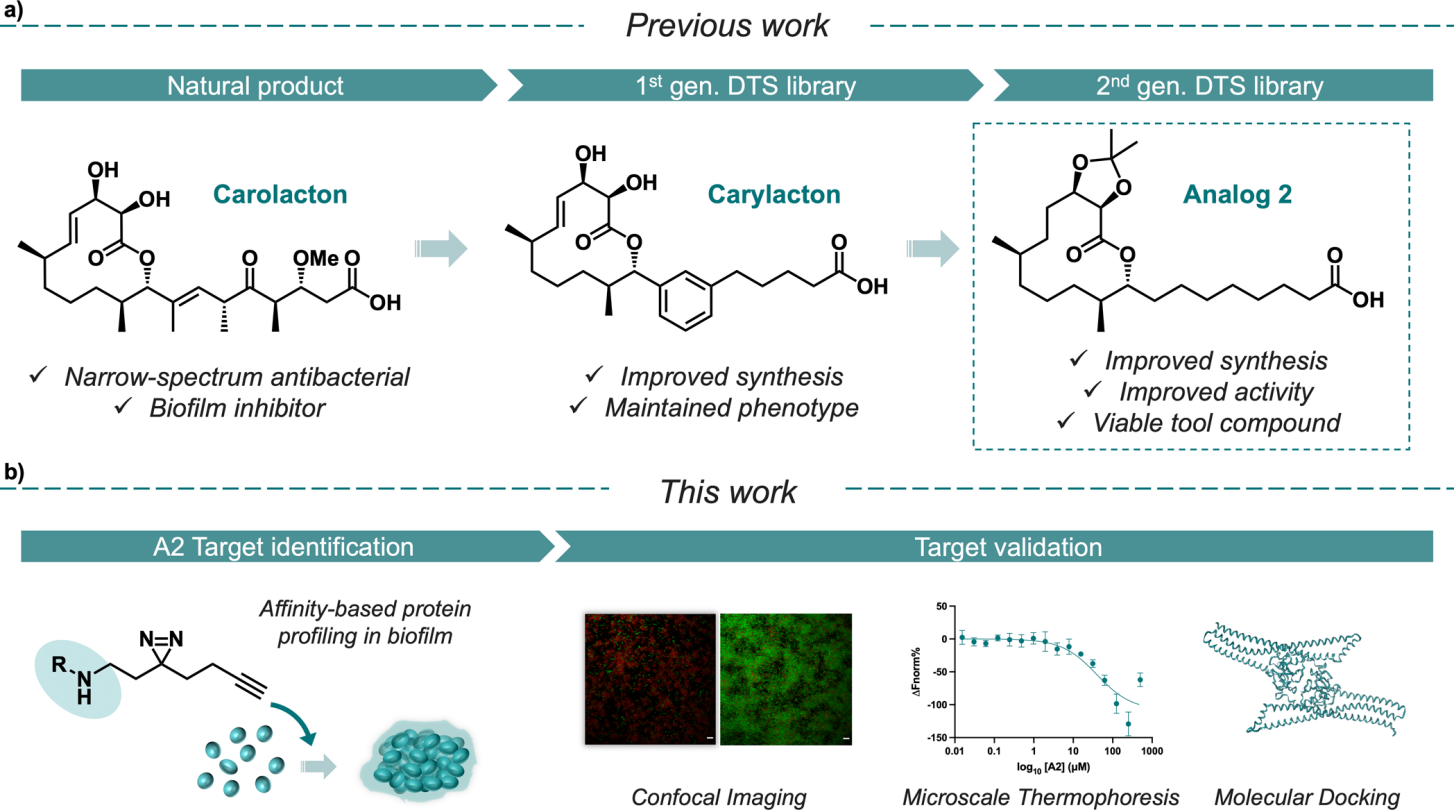
(a) Natural product inspiration and simplification led to the discovery of **A2**. (b) The terminal carboxylic acid was used to append the minimalist probe to access a photo-cross-linking probe, **A2-PP**, which was used for biological target identification.

Carolacton is a natural product biosynthesized by *Sorangium cellulosum* and has been shown to affect carbon utilization, cell wall biosynthesis, amino acid metabolism, and biofilm formation in *Streptococcus mutans*.^24−30^ Demonstration of activity against *S. pneumoniae* alluded to a conserved streptococcal target. Subsequently, Müller and co-workers reported that carolacton targets folate dehydrogenase (FolD) after selecting for a resistant mutation in this gene using an *Escherichia coli* efflux knockout (*E. coli* Δ*tolC)*.^31^ This finding was further corroborated in an *in vitro* biochemical assay using repurified FolD. However, to date FolD has not been linked to any cell wall or biofilm processes. To leverage the appealing biological activity of carolacton, while reducing the number of synthetic steps, we previously employed two rounds of DTS (Figure 1a). From these efforts emerged a simplified compound, Analog 2 (**A2**), that displayed improved growth inhibition, killing, and antibiofilm activity when tested against the oral pathogen *Streptococcus mutans*.^14^ Using a genetic knockout screen, we connected **A2** to a master regulator in cell division and biofilm formation pathways, ccpA, but the molecular target has remained elusive. Leveraging the simplified scaffold, we synthesized a photoaffinity probe for affinity-based protein profiling (AfBPP) in a biofilm-inducing environment (Figure 1b). The subsequent cross-linking proteomic experiments identified a small subset of potential protein targets, which after validation, uncovered glucan binding protein B (GbpB), a putative peptidoglycan hydrolase, as the target of **A2**. GbpB plays an essential role in cell wall septal division and is highly conserved across the streptococcus genus, providing a novel strategy to prevent pathogenic streptococcal infections.^32,33^ We report the discovery of a first-in-class binder of the essential peptidoglycan hydrolase, GbpB/PcsB.

## Results

### Unlike Carolacton **A2** Does Not Inhibit FolD

Due to the synthetic similarity of carolacton and **A2**, we wanted to first determine if **A2** also inhibited FolD. Bacterial growth assays and confocal laser microscopy were conducted using an *S. mutans* FolD knockout strain and a WT strain. The effect of **A2** on bacterial growth and biofilm morphology was unchanged between the two strains, which likely excludes FolD as the responsible player behind the observed phenotype (Figures S1, S2). **A2** may not interact with FolD due to limited access to the cytosol, therefore, we conducted a dehydrogenase assay using purified *E. coli* FolD as previously described.^31^ Under these assay conditions, carolacton exhibited an IC_50_ value of 94 nM, but **A2** was inactive up to 10 μM (Figure S3). This evidence demonstrates that the effect against *S. mutans* biofilms imparted by the simplified scaffold is likely not related to FolD inhibition, and the natural product and **A2** have, at least in part, different targets.

### Affinity-Based Protein Profiling Identifies GbpB as the Biological Target of **A2**

**Figure 2:**
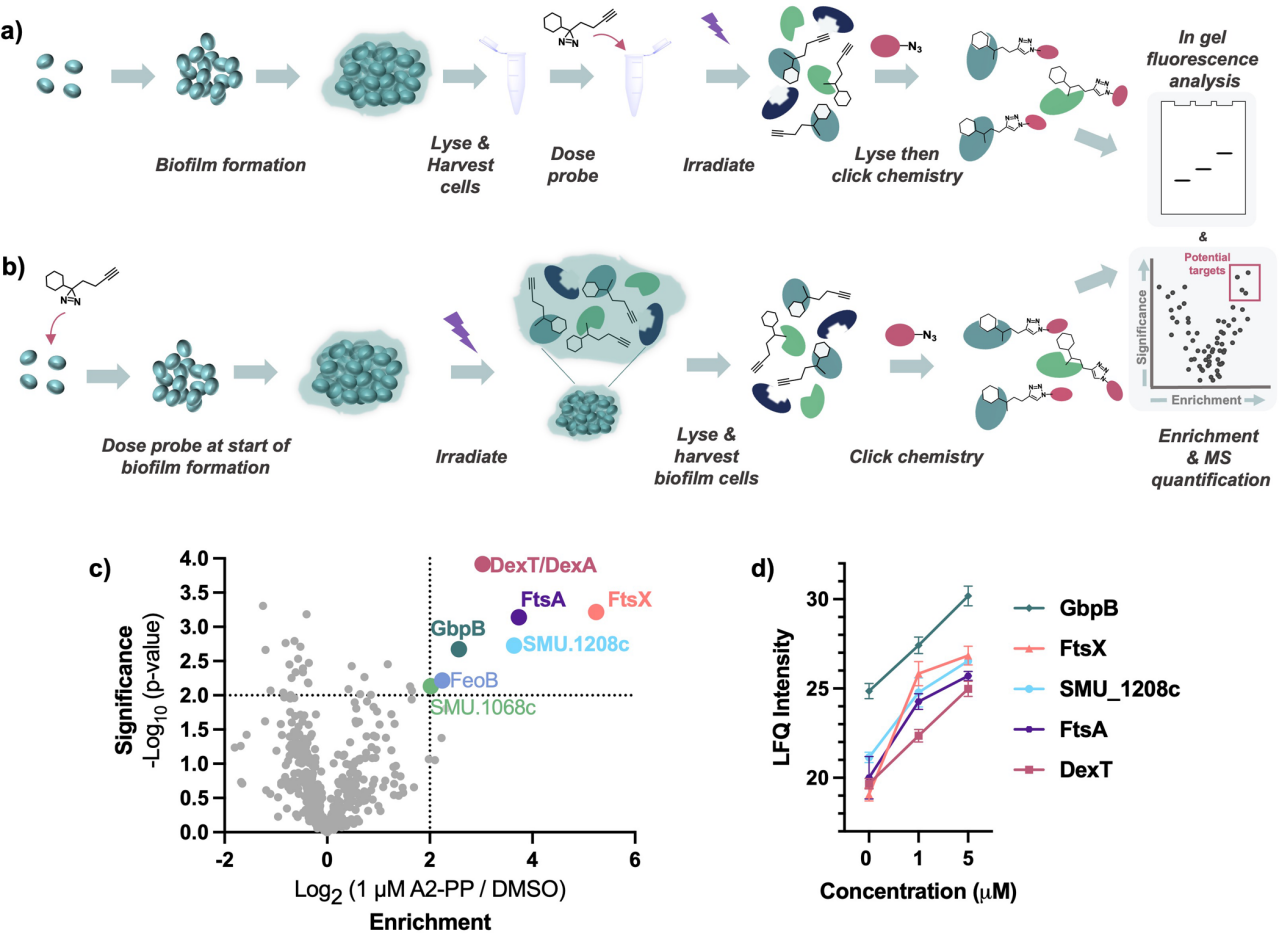
Workflow for affinity-based protein profiling using mature biofilm cells (a) and in situ biofilm affinity-based protein profiling (b). **A2-PP** is incubated either with mature or growing biofilms, irradiated, and harvested. After cell lysis, click chemistry is performed with rhodamine-N_3_ or biotin-N_3_ enabling either direct in-gel fluorescence analysis, or enrichment for MS quantification, respectively. (c) AfBPP experiment using **A2-PP** in *S. mutans* biofilm compared to DMSO control. (d) Dose-dependent Label-Free Quantification intensity (two-sided two-sample *t* test, *n* = 3 independent experiments per group).

We have had previous success using AfBPP to identify the targets of active compounds.^34−36^ We leveraged the terminal carboxylic acid as a handle to develop a chemical probe for AfBPP experiments (Figure 1b). We synthesized **A2**-photoprobe (**A2-PP**) that contained a minimalist photoaffinity handle with a photoexcitable diazirine and an alkyne handle for enrichment and analysis. **A2-PP** comes directly from **A2** in 61% yield using standard amidation conditions (Figure S4). We were interested in exploring AfBPP using both mature and developing biofilms. In the former, cells are harvested prior to dosing in the photoprobe (Figure 2a), whereas in the latter, the photoprobe is dosed at the beginning of growth (Figure 2b). We observed a higher level of protein enrichment when we incubated **A2-PP** with developing *S. mutans* biofilms, in line with the observed inhibitory phenotype, thus we optimized based on this workflow. SDS-PAGE analysis revealed a dose-dependent protein cross-linking response, and optimal concentrations were chosen in the range of 1 and 5 μM (Figure S5). After following the workflow illustrated in Figure 2b, the enriched proteins were digested with trypsin and analyzed by LC-MS/MS. Label-free quantification (LFQ) was used to calculate the protein enrichment using **A2-PP**.^37^ Proteins were considered targets only if they were enriched at 1 μM **A2-PP** (Figure 2c) and exhibited a dose-dependent enrichment upon exposure to 5 μM **A2-PP** (Figures 2d, S6, S7). Our analysis identified seven putative targets (Figure 2c), but the dose-dependent response (Figure 2d) supported only five of the seven as true interactions. DexA and GbpB exhibited the most significant dose-dependent responses, with the most prominent enrichment observed with GbpB (Figure 2d). Interestingly, in other *Streptococcus* spp., FtsA and FtsX have been shown to participate in late-stage cell division in a complex with PcsB, a GbpB homologue.^38−42^ This connection has yet to be shown in *S. mutans*, but our results suggest this relationship is also likely.

We sought to determine which of the five candidate proteins is the primary target of **A2**. We attempted to develop **A2**-resistant strains of *S. mutans* through serial passage assays, but our efforts were inconclusive. Alternatively, we once again employed a screen utilizing the Quivey *S. mutans* knockout library^43^ where strains lacking the target of **A2** should be resistant to compound treatment. We grew Δ*ftsA*, Δ*dexT*, Δ*smu.1208c*, and Δ*ftsX* with a serial dilution of **A2** and compared the results to WT UA159. We found that deletion of *ftsA*, *dexT*, or *smu.1208c* did not rescue bacterial growth or biofilm formation, suggesting that the gene products are not the direct targets of our compound (Figure S8). We specifically had to test the *ftsX* mutant under biofilm-promoting conditions and found that although **A2** still demonstrated activity, it varied at high concentrations (Figure S9). To investigate this further, we analyzed the Δ*ftsX* biofilm after treatment with **A2** using LIVE/DEAD stain. The biofilm IC_50_ was recorded at 44 μM, and we therefore tested Δ*ftsX* at 63 μM to visualize phenotypic changes.^14^ The untreated Δ*ftsX* biofilm appears robust and healthy. However, when treated with **A2**, we observed a decrease in biofilm formation/attachment and very few live cells (Figure S9). These data suggest that the absence of *FtsX* enhances the killing and antibiofilm effects of **A2**.

**Figure 3:**
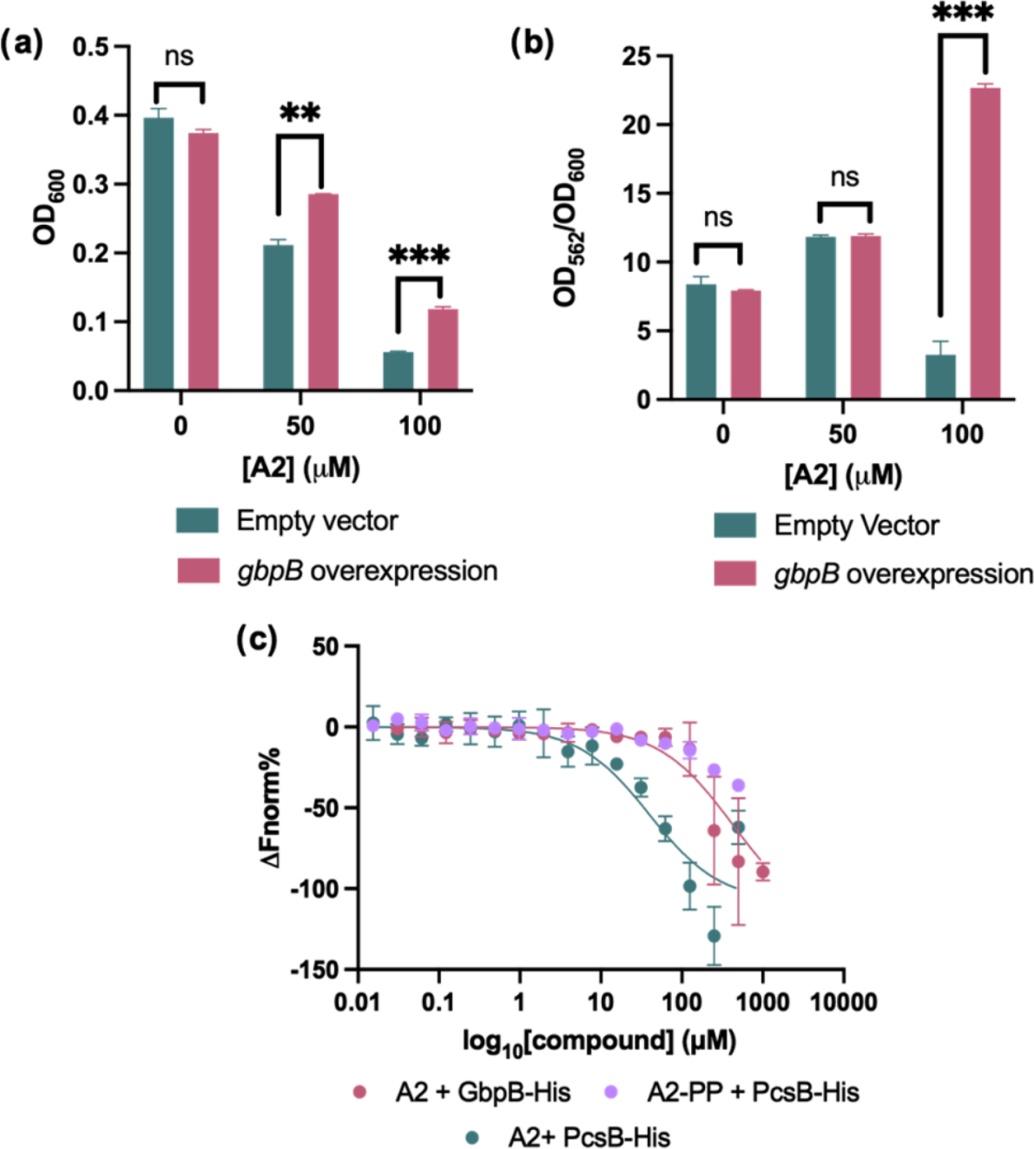
(a) Growth inhibition of A2-treated bacterial cells in *gbpB* overexpression strain and empty vector strain at 16h. Data represents two biological replicates (+ s.d.). *p* value: ns = not significant; ** ≤ 0.01, *** < 0.002; two-tailed Student’s *t* test; (b) Biofilm formation of A2-treated biofilm cultures in *gbpB* overexpression strain and empty vector strain at 16h. Biofilm formation was normalized to account for differences in bacterial growth by dividing OD562/OD600. Data represents two biological replicates (+ s.d.). *p* value: ns = not significant; *** ≤ 0.002; two-tailed Student’s *t* test; (c) Microscale thermophoresis using RED-tris-NTA-labeled protein with **A2** or **A2-PP**. Data represents the average of 3 independent measurements, error bars represent the standard deviation.

The remaining proteomic lead, GbpB, did not have a knockout strain available as it is essential for growth. Instead, we overexpressed GbpB to test whether it conferred resistance to **A2** treatment.^15^ We constructed a pVPT-GbpB plasmid and transformed it into *S. mutans* UA159 alongside a strain containing an empty vector that was used as a negative control.^44^ Incubation of the overexpression strain with 150 μM of **A2** is sufficient to inhibit growth (Figure 3a). However, *gbpB* overexpression was protective against compound treatment at 50 μM and 100 μM. Similarly, the strain overexpressing *gbpB* demonstrated drastically improved biofilm formation compared to WT when treated with 100 μM of **A2** (Figure 3b). We then used Microscale Thermophoresis (MST) to evaluate the binding of **A2** and GbpB. We purified GbpB with a C-terminal His-tag to facilitate purification and MST analysis and found that A2 exhibited a dissociation constant of 391 ± 128 μM (Figures 3c, S11). We hypothesize that the weak binding is because GbpB typically functions in a complex with FtsX and FtsE. This would explain why we see activities at lower concentrations but only see moderate binding *in vitro* with purified enzyme. Although further investigation is needed, our AfBPP results suggest that **A2** is in proximity to FtsX thus supporting our hypothesis.

### **A2** Binds the CHAP Domain of GbpB and PcsB

**Figure 4:**
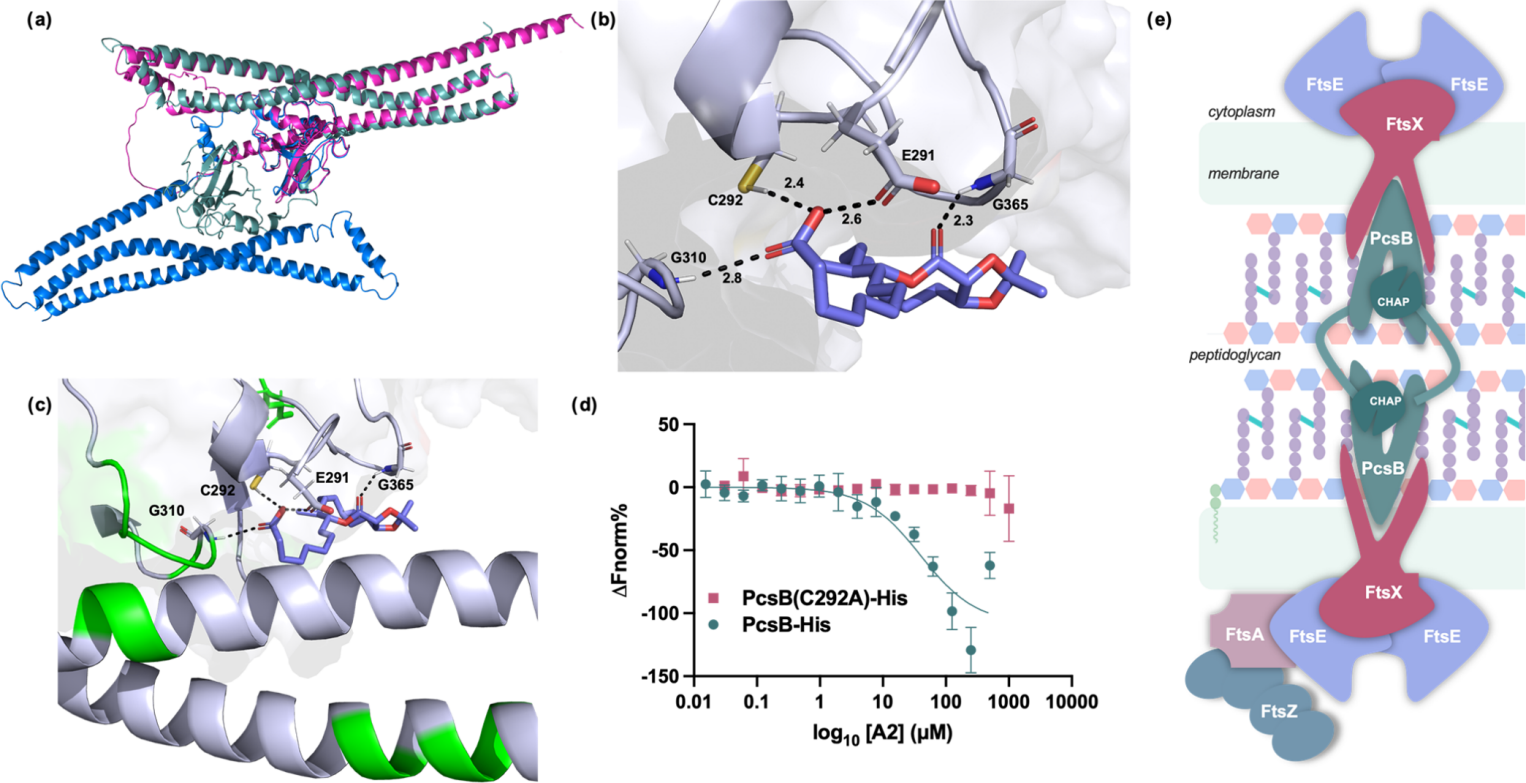
(a) Overlay of *spn*PcsB dimer (blue and teal; PDB: 4CGK) with *smu*GbpB monomer (pink) that was solved using AlphaFold. (b) Computationally predicted docking pose between **A2** and *spn*PcsB CHAP domain. (c) Computationally predicted docking pose between **A2** and *spn*PcsB CHAP domain with cross-linking sites by **A2-PP** depicted in green. (d) Microscale thermophoresis of RED-tris-NTA-labeled proteins with A2. Data represents the average of 3 independent measurements, error bars represent the standard deviation. (e) Schematic of inactive PcsB dimer in complex with other members of the divisome.

In *S. mutans*, GbpB has been implicated primarily in biofilm formation, while its role in bacterial growth is poorly understood.^32,33,45^ Sequence analysis revealed that GbpB is comprised of a leucine zipper, a long linker domain, and a conserved C-terminal CHAP (cysteine, histidine-dependent, amidohydrolase/peptidase) domain. The GbpB CHAP domain shares high sequence similarity with N-acetylmuramoyl-l-alanine amidases/endopeptidases in various *Streptococcus* spp. (Figure S13) that are responsible for septum cleavage. The best characterized of these homologues is PcsB derived from *S. pneumoniae*, and it is the only homologue with a crystal structure.^38−41^ To compare the structural features of GbpB to PcsB, we used AlphaFold2 to predict the structure of *smu*GbpB, and found that they were highly homologous (Figures 4a, S14–17).

To date, there are no known binders or inhibitors of the CHAP domain. For these reasons, we were interested in further characterizing the binding interaction of **A2**. We proceeded with PcsB, as we could utilize the structural data from the Protein Data Bank (PDB: 4CGK) to aid our analyses.^38^ We expressed and purified two *spn*PcsB constructs, one with the His_6_-tag at the N-terminus (*spn*His-PcsB) and one with the His_6_-tag at the C-terminus (*spn*PcsB-His) to use in MST binding experiments. **A2** exhibited a *K*_d_ of 40 ± 9 μM for PcsB-His (Figure 3c, S18). On the contrary, we tested **A2** with His-PcsB and found that the *K*_d_ increased to 312 ± 51 μM, demonstrating that the PcsB-His is the better construct for our binding experiments (Figure S19). Additionally, we observed that carolacton does not bind PcsB at concentrations lower than 1 mM, supporting that carolacton and **A2** have different targets (Figure S22). Concurrently, we used the Glide docking module of Maestro (Schrödinger suite) to predict the binding mode of **A2** and **A2-PP**. In two of the top-scoring poses with **A2-PP**, the diazirine is placed near the entrance of the catalytic site (Figure S23). In the next-highest scoring pose, the elongated side chain in **A2-PP** is placed peripheral to the catalytic site (Figure S23). In the computationally predicted pose, the alkyl chain is pointing into the catalytic pocket, priming the terminal carboxylic acid for hydrogen bonding interactions with Gly310, Glu291, and Cys292 (Figure 4b). In addition, the lactone carbonyl moiety also interacts with Gly365. The interactions observed with essential members of the catalytic triad support a binding model where **A2** blocks the catalytic domain. The importance of the carboxylic acid proton in the binding model provides rationale for the enhanced activity against *S. mutans* and is further supported by our previous DTS campaigns.^6,14,29^

To corroborate the MST binding data, we also incubated purified *spn*PcsB-His or *spn*His-PcsB with **A2-PP**. After irradiation and enrichment, intact protein analysis was carried out via ESI-qTOF-MS and a mass increase corresponding to **A2-PP** cross-linking was observed between *spn*PcsB-His but not *spn*His-PcsB (Figures S24, 25), suggesting a specific binding interaction as seen previously with MST. In both its monomeric and dimeric forms, PcsB exists in an autoinhibitory conformation, wherein the catalytic CHAP domain is tucked into the N-terminus, blocking the active site. Typically, substrate accessibility is facilitated by FtsX. We postulate that when the His-tag is placed at the C-terminus, it can disrupt the stabilizing interactions between the two domains and allows the probe access to the active site. To map the binding site, the cross-linked samples were also subjected to proteolytic digestion with trypsin and analyzed with LC-MS/MS. Analysis of the protein cross-linking sites revealed localization peripheral to the CHAP domain of PcsB (Figure 4c). To directly test the binding model, we purified a mutated version of *spn*PcsB-His [PcsB(C292A)-His], where the catalytic cysteine is mutated to an alanine, since the docking model suggested important hydrogen bonding interactions with this residue. We found that this mutation of the catalytic cysteine was sufficient to eliminate binding when measured with MST (Figures 4d, S26). Collectively, these data suggest that **A2** binds the CHAP domain of PcsB, making it the first small molecule binder of this domain.

### **A2** Is a Narrow-Spectrum *Streptococci-*Specific Inhibitor

The essential and conserved nature of the target of **A2**, GbpB/PcsB, prompted us to explore our compound’s utility as a general streptococci inhibitor. We screened **A2** against a panel of pathogenic and commensal *Streptococcus* species., including *S. pneumoniae*, *Streptococcus pyogenes* (Group A Streptococcus, GAS), *S. alagactiae* (Group B Streptococcus, GBS), *Streptococcus gordonii* and *Streptococcus sanguinis* (Figure S28). **A2** exhibited an MIC of 125 μM across all strains and an IC_50_ between 29 and 62 μM, which is in close agreement with the activity against *S. mutans*.^14^

## Discussion

Herein, we uncover the protein target for the natural product-inspired compound, **A2**, which was discovered through the synthetic exploration of carolacton. Using a *in situ* AfBPP approach in biofilm cultures, we successfully identified five putative targets. Gene deletion, overexpression, and binding studies identified GbpB as the molecular target, which is essential and conserved across the Streptococcus genera. It has been implicated in the early steps of biofilm formation and its essentiality is due to its role in cell wall septum cleavage. In the latter role, it is purported to function in a complex that contains two of the other AfBPP hits, FtsA and FtsX (Figure 4e). We used the best characterized homologue of GbpB, PcsB, to determine the binding site of **A2**. Molecular docking studies identified a binding site in the CHAP domain, the catalytic site of the protein, which was validated using *in vitro* cross-linking and MST experiments. We then screened a panel of *Streptococcus* strains and found that activity was retained across the different species. Although we were unable to use the true target, GbpB, for our validation studies, our results show that **A2** promotes cell death through its interaction with GbpB/PcsB.

To the best of our knowledge, this is the first example of *in situ* biofilm AfBPP and the first small molecule binder of a bacterial CHAP domain. As the GbpB/PcsB cell wall complex is further characterized, our understanding of the precise mechanism of **A2** will only become clearer, as an inhibition assay of this complex is not yet available. This study lays the foundation for additional rounds of medicinal chemistry to improve potency, chemical probe design, and investigation into this class of essential cell wall CHAP hydrolases. Application of these tools can illuminate the biochemistry underlying cell wall division and biofilm formation in Gram-positive streptococci and facilitate the discovery of methods to circumvent current antibiotic resistance mechanisms.

## Supporting Information Available

Assay procedures, compound characterization and associated spectral images (PDF)

## Abbreviations

AfBPP: affinity-based protein profiling
GbpB: glucan binding protein B
MST: microscale thermophoresis
CHAP: cysteine, histidine-dependent, amidohydrolase/peptidase domain
*smu*: *Streptococcus mutans*
*spn*: *Streptococcus pneumoniae*
