# Supplementary material for: In Situ Biofilm Affinity-Based Protein Profiling Identifies the Streptococcal Hydrolase GbpB as the Target of a Carolacton-Inspired Chemical Probe
Source: J Am Chem Soc. 2024 Aug 12;146(33):23449–56. doi: 10.1021/jacs.4c06658 (PMC11345752; doi:10.1021/jacs.4c06658)
Supplement: Supplementary file 1 — ja4c06658_si_001.pdf [file ja4c06658_si_001.pdf]

# Supporting Information

## In situ biofilm affinity-based protein profiling identifies the streptococcal hydrolase GbpB as the target of a carolacton-inspired chemical probe

Amber M. Scharnow[‡, † 1], Amy E. Solinski[‡, † 1], Sebastian Rowe[2], Ines Drechsel, Hua Zhang[4], Elana Shaw[†,1], Julia E. Page[5], Hui Wu[†,4], Stephan A. Sieber[3], William M. Wuest[1]*

[1]*Department of Chemistry, Emory University, Atlanta, GA 30322 USA.*

[2]*Department of Chemical Biology, Harvard University, Cambridge, MA 02138, USA.*

[3]*Department of Chemistry, Center for Functional Protein Assemblies, Technical University of Munich, Garching D-85747, Germany.*

[4]*Department Departments of Pediatric Dentistry, Microbiology, Schools of Dentistry and Medicine, University of Alabama at Birmingham, Birmingham, 35294, Alabama, USA*

[5]*Department of Microbiology, Blavatnik Institute, Harvard Medical School, Boston, MA 02115*

***Supplementary Figures*** ........................................................................................ ***3***
***Chemical Methods*** ................................................................................................ ***18***
***Biological Methods*** .............................................................................................. ***18***
***References*** ......................................................................................................... ***23***
***Compound spectra*** ................................................................................................ ***24***

## Supplementary Figures

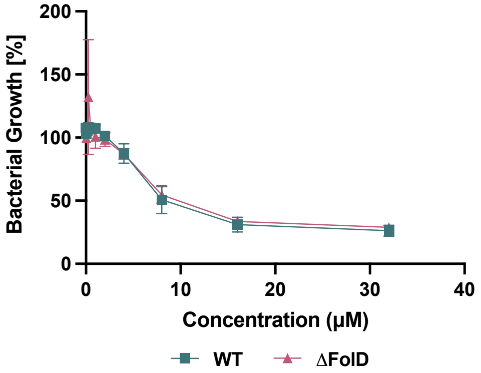


**Figure S1** Growth curves of **A2**-treated strains in Todd Hewitt Broth (pH = 5.5). Data shown represents biological triplicate. Data is normalized to cell growth without any treatment.

.

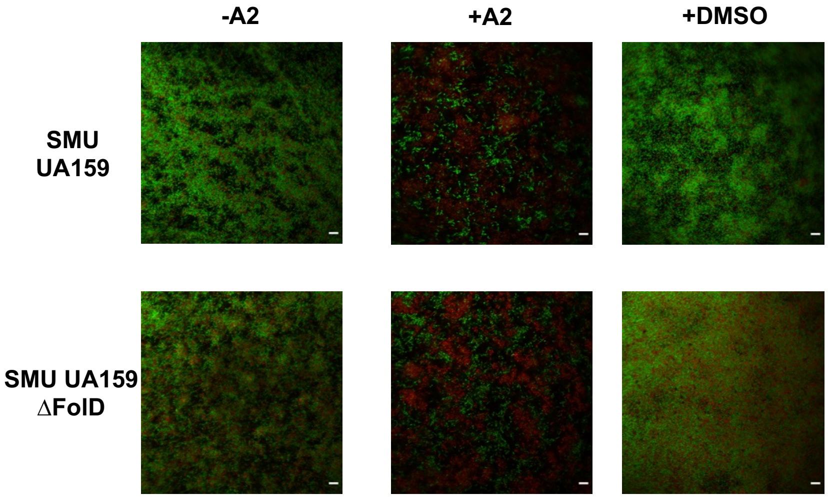


**Figure S2** Confocal Microscopy images were taken with a 60X objective, and LIVE/DEAD stain was used to visualize cell viability. Compound was tested at 63 µM. Scales bars are 10 µm.

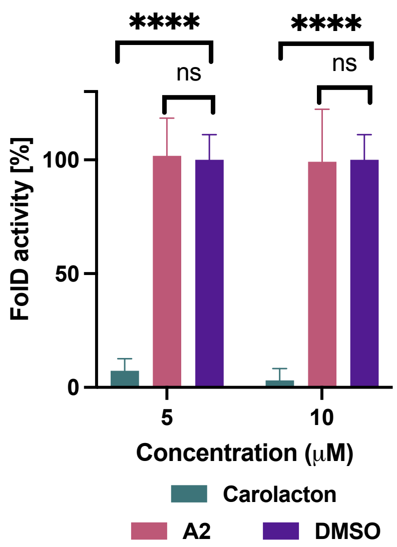


**Figure S3** Folate dehydrogenase assay. The effect of carolacton and A2 on FolD activity were compared to the DMSO vehicle control at 5 and 10 μM. *p value: ns = not significant; **** = < 0.0001; two-tailed Student's t-test;*

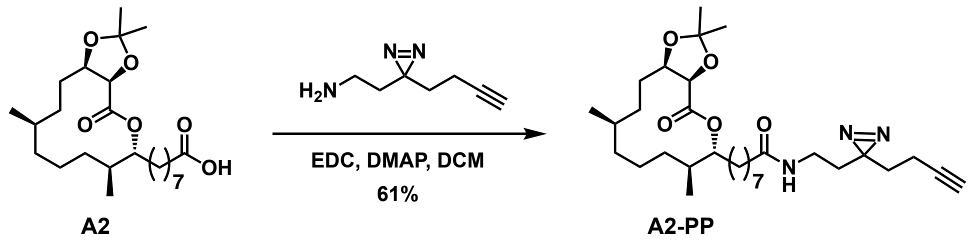


**Figure S4** Synthetic scheme for the amidation conditions used for the synthesis of A2-PP.

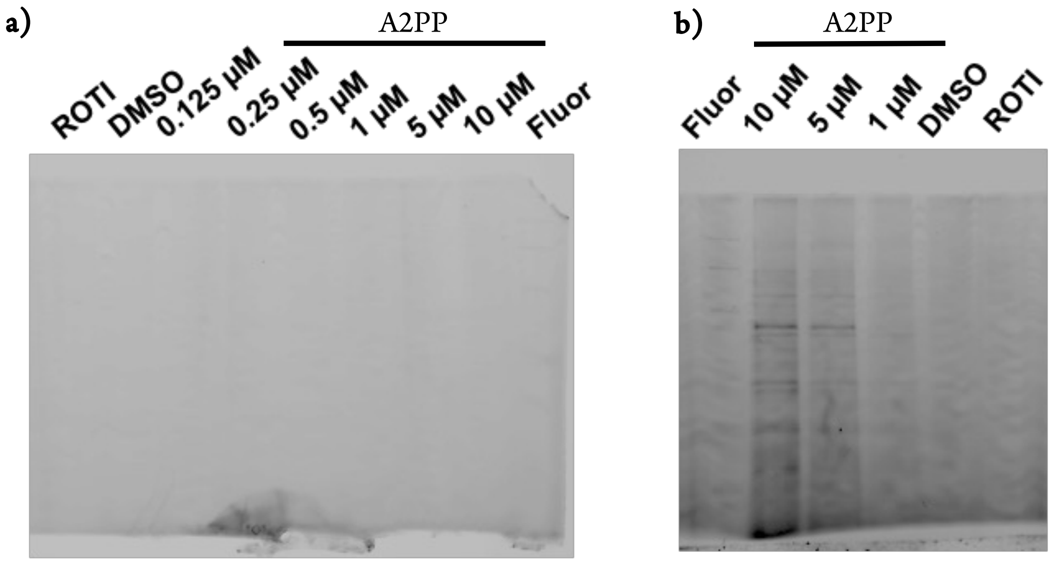


**Figure S5** Gel-based Affinity-based Protein Profiling. **a)** SDS-page gel showing crosslinking efficiency of analog 2-probe following incubation with mature biofilm cells. Roti = RotiMark and Fluor = Fluorescence marker are used as markers for protein size. **b)** SDS-page gel showing crosslinking efficiency of analog 2-probe following incubation with actively growing biofilm cells. Roti = RotiMark and Fluor = Fluorescence marker are used as marker for protein size.

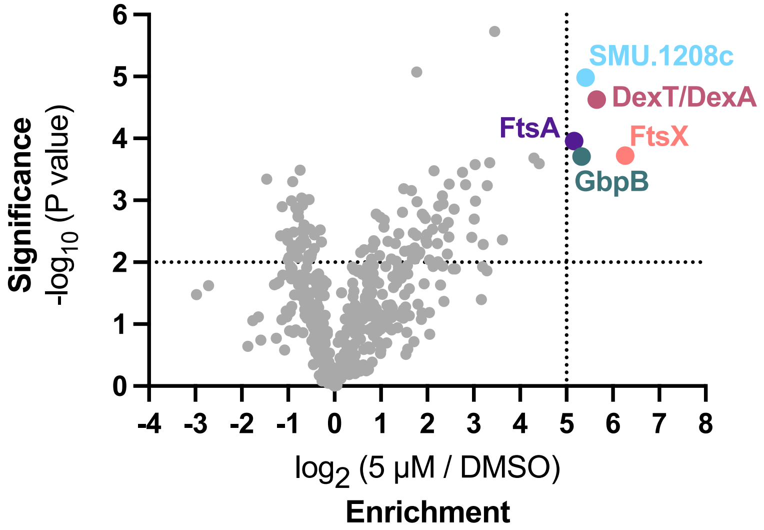


**Figure S6** AfBPP experiment using **A2-PP** in *S. mutans* biofilm. Volcano plot shows a $\log_2$ -fold enrichment of proteins after treatment with **A2-PP** in (5 µM) compared with DMSO. The vertical and horizontal threshold lines represent a $\log_2$ -fold enrichment ratio of 5 and a $-\log_{10}$ P value of 2 (two-sided two-sample t-test, n = 3 independent experiments per group), respectively.

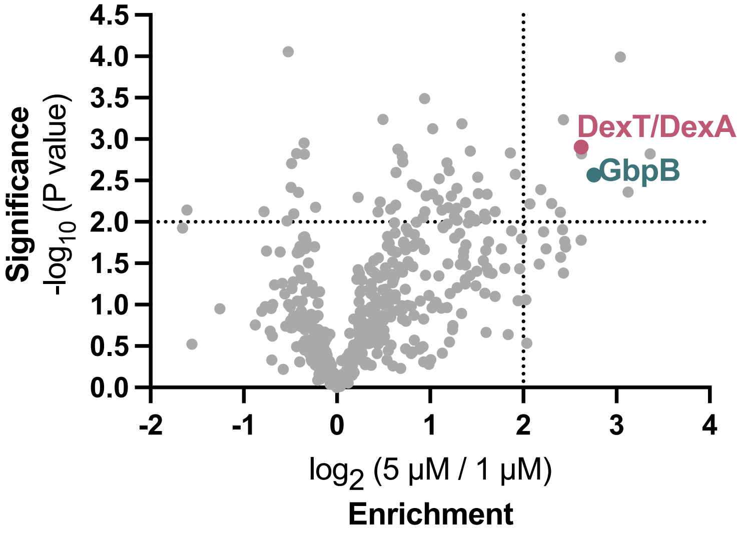


**Figure S7** AfBPP experiment using **A2-PP** in *S. mutans* biofilm. Volcano plot shows a $\log_2$ -fold enrichment of proteins after treatment with **A2-PP** in (5 μM) compared with A2-PP (1 μM). The vertical and horizontal threshold lines represent a $\log_2$ -fold enrichment ratio of 2 and a $-\log_{10}$ P value of 2 (two-sided two-sample t-test, n = 3 independent experiments per group), respectively.

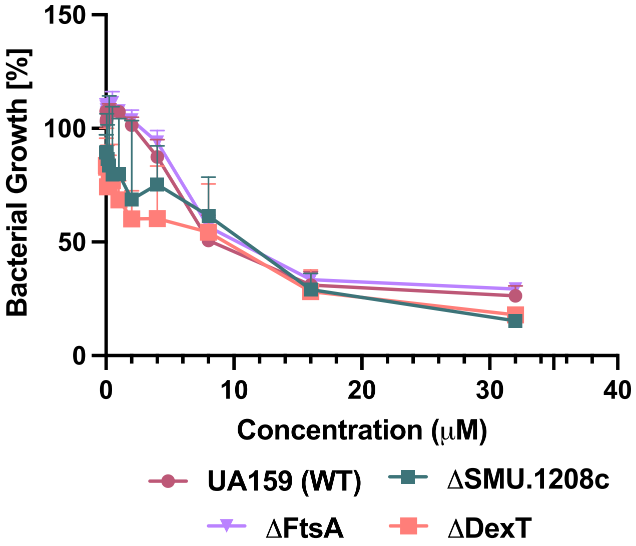


**Figure S8** Growth curves of **A2**-treated strains in Todd Hewitt Broth (pH = 5.5) after 22 hours of incubation. Data shown represents biological triplicate. Data is normalized to cell growth without any treatment. Cells were grown under these conditions to conserve compound, as the compound is most active at pH 5.5.

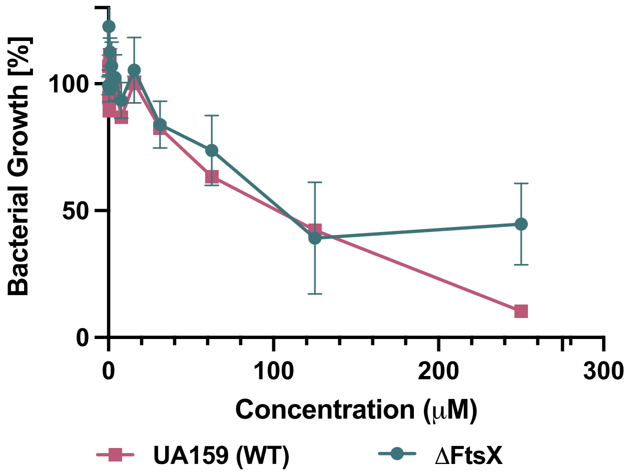


**Figure S9** Growth curves of **A2**-treated strains in Todd Hewitt Broth supplemented with 0.1% sucrose (w/v) (THBS)) after 22 hours of incubation. Data shown represents biological triplicate Data is normalized to cell growth without any treatment. Cells were grown under these conditions because this mutant does not grow in pH 5.5 media.

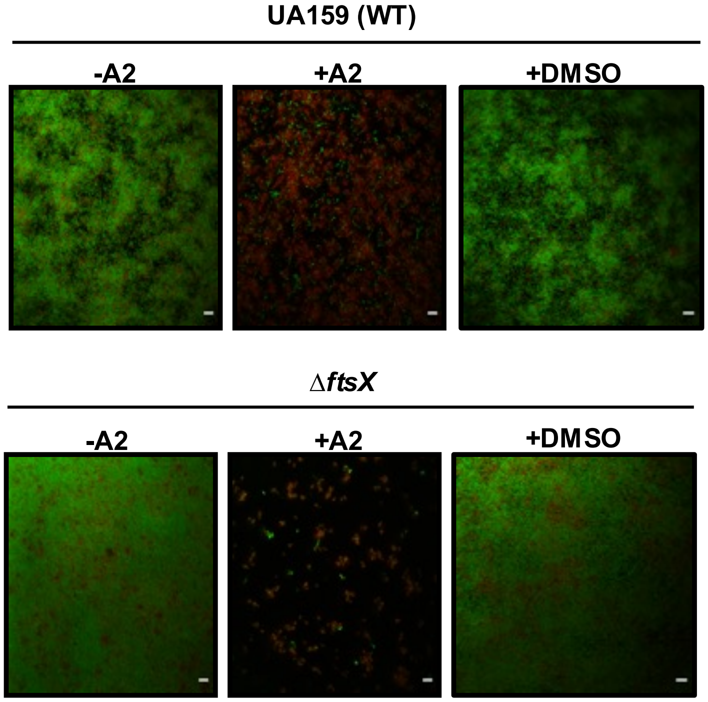


**Figure S10** Confocal laser microscopy images of UA159 WT cells or Δ*ftsX* treated with A2 (63 μM) dissolved in DMSO and stained with LIVE/DEAD. Images were taken at 10X objective and scale bars are 10 μM.

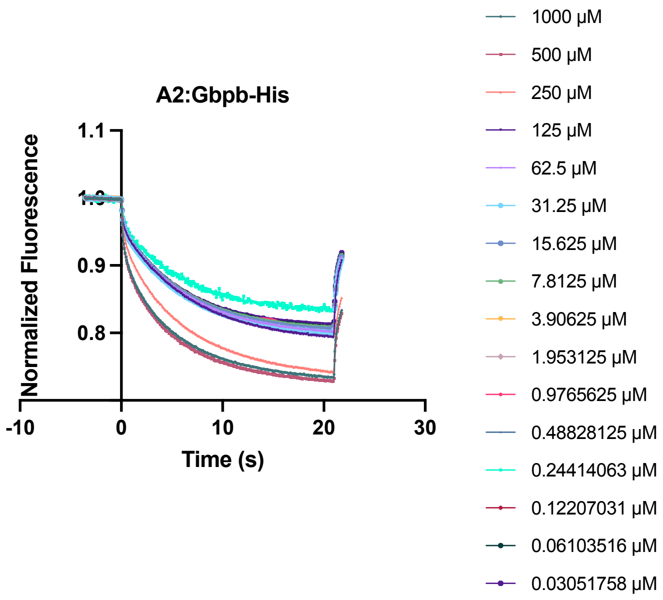


**Figure S11** Microscale thermophoresis traces of GbpB-His and A2.; a) In this MST experiment the concentration of the RED-tris-NTA-labeled GbpB-His was kept constant (10 nM), while the concentration of the non-labeled binding partner (A2) was varied between 0.015 µM – 500 µM or 1000 µM. After 10 min incubation the samples were loaded into Monolith NT.115 Premium Capillaries (NanoTemper Technologies) and the MST measurement was performed using the Monolith NT.115Pico (NanoTemper Technologies) at 20 % LED power and medium MST power. An MST-on time of 15 s was used for analysis (n = 3 independent measurements, error bars represent the standard deviation). . b) MST traces recorded for this experiment.

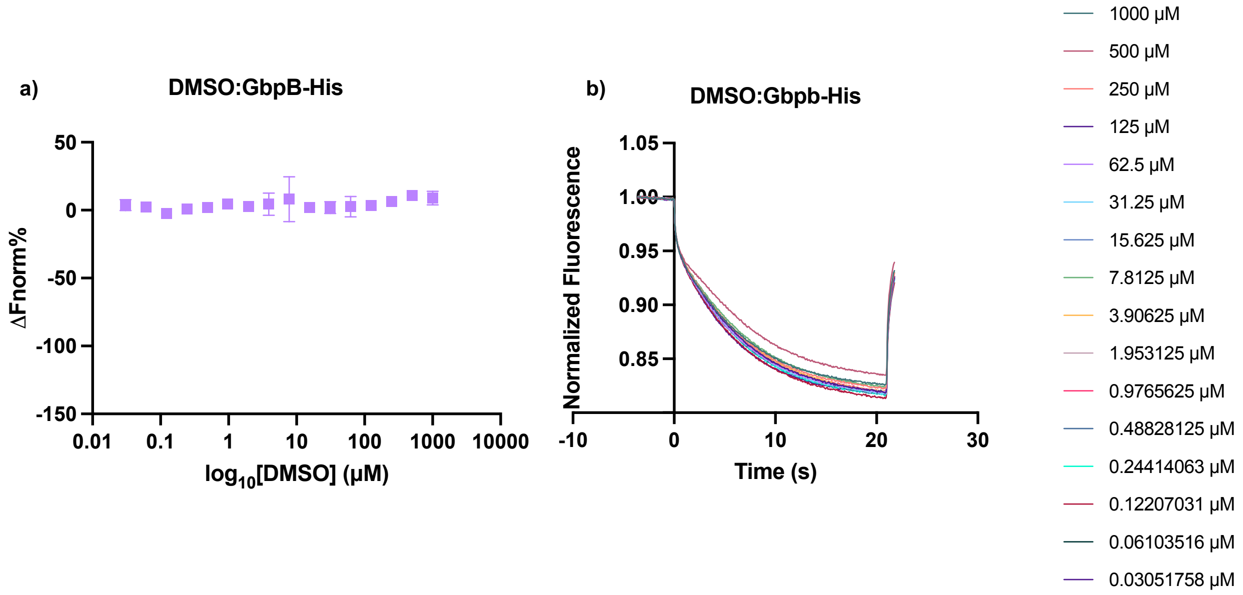


**Figure S12** Microscale thermophoresis of PcsB-His + DMSO.; a) In this MST experiment the concentration of the RED-tris-NTA-labeled GbpB-His was kept constant (10 nM), while the concentration of the non-

labeled binding partner (A2) was varied between 0.015 µM – 500 µM or 1000 µM. After 10 min incubation the samples were loaded into Monolith NT.115 Premium Capillaries (NanoTemper Technologies) and the MST measurement was performed using the Monolith NT.115Pico (NanoTemper Technologies) at 20 % LED power and medium MST power. An MST-on time of 15 s was used for analysis (n = 3 independent measurements, error bars represent the standard deviation). . b) MST traces recorded for this experiment.

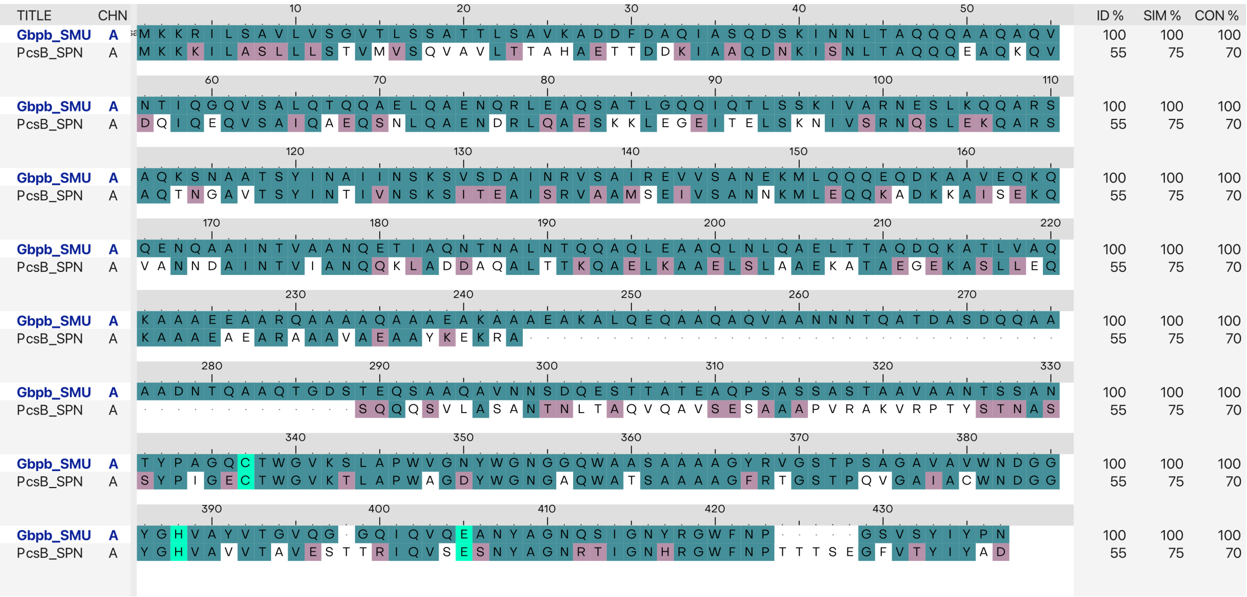


**Figure S13** Alignment between the *smu*GbpB and *spn*PcsB. Alignment was constructed in Maestro Multiple sequence viewer and analyzed with ClustalW. Dark teal residues are identical, maroon residues are similar, and the catalytic triad is highlighted in light green.

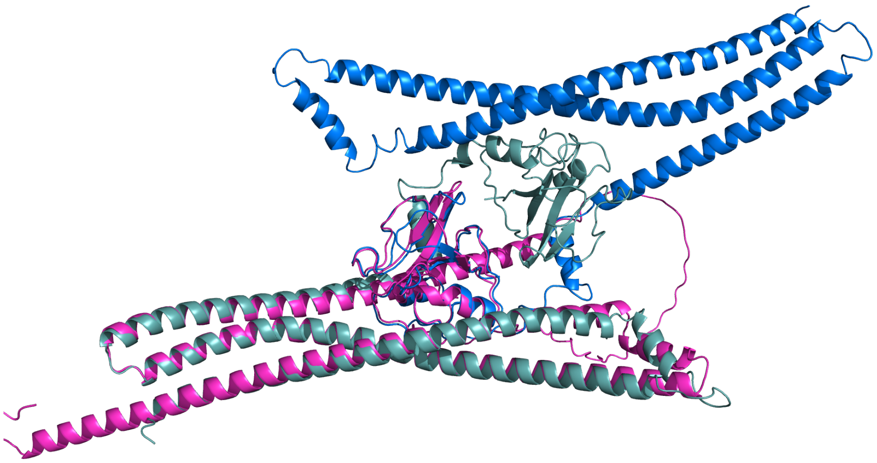

**Figure S14** Overlay of *spn*PcsB dimer (blue and teal; PDB:4CGK) with *smu*GbpB monomer (pink) that was solved using AlphaFold.

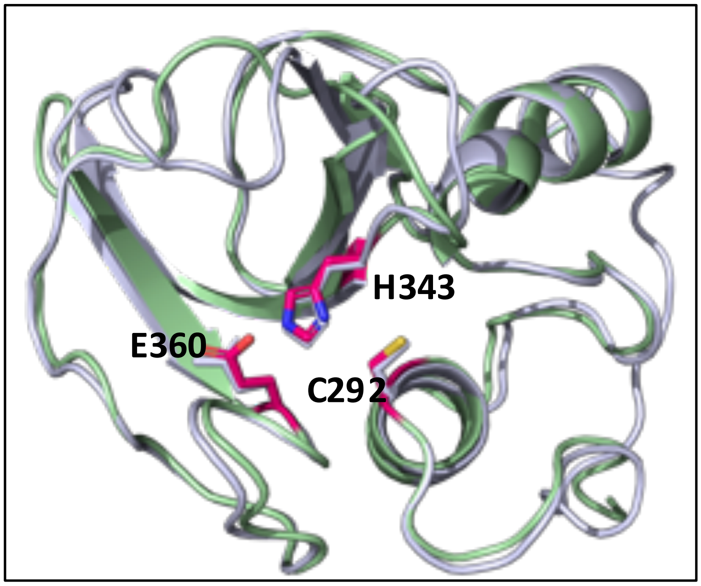


**Figure S15** Overlay of *spn*PcsB CHAP domain (grey; PDB:4CGK) with *smu*GbpB CHAP domain (green) that was generated using AlphaFold. The catalytic triad is highlighted in pink.

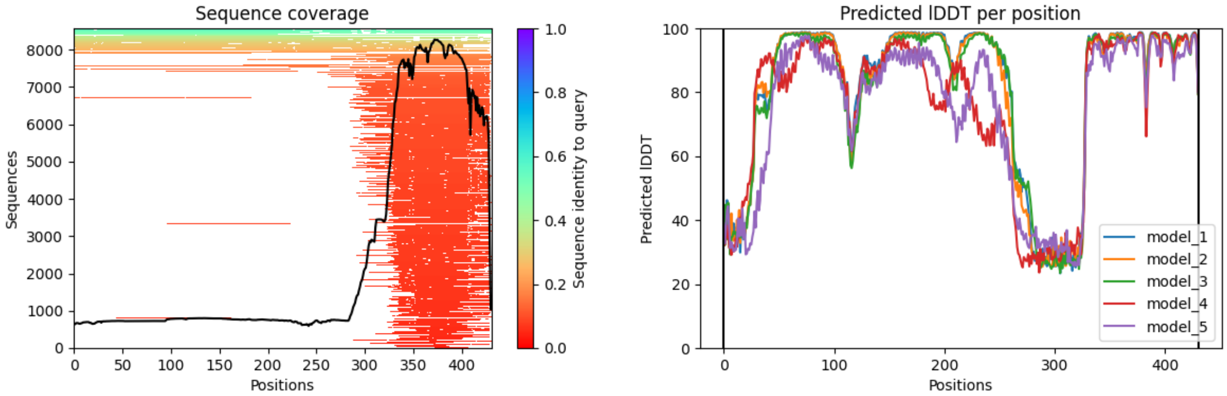


**Figure S16** AlphaFold2 sequence coverage and predicted IDTT (per-residue confidence metric)

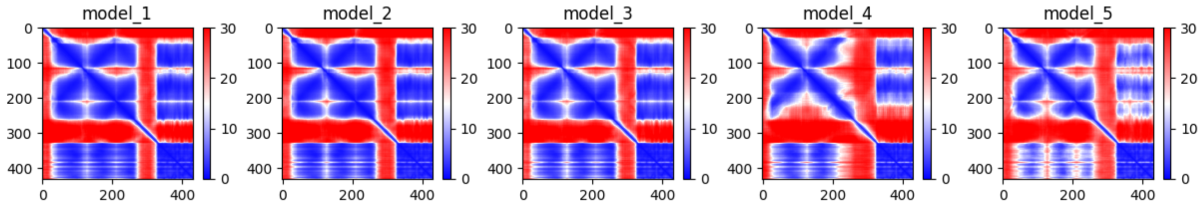


**Figure S17** AlphaFold2 Predicted alignment error (PAE)

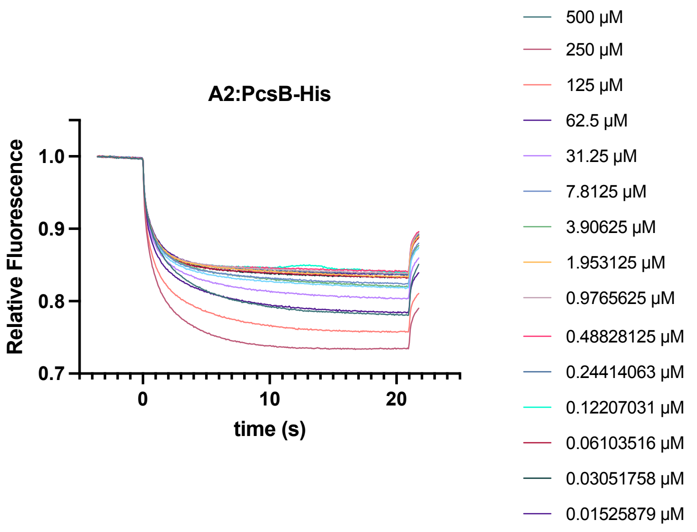


**Figure S18** Microscale thermophoresis of PcsB-His + A2; a)In this MST experiment the concentration of the RED-tris-NTA-labeled Pcsb-His was kept constant (10 nM), while the concentration of the non-labeled binding partner (A2) was varied between 0.015 µM – 500 µM or 1000 µM. After 10 min incubation the samples were loaded into Monolith NT.115 Premium Capillaries (NanoTemper Technologies) and the MST measurement was performed using the Monolith NT.115Pico (NanoTemper Technologies) at 20 % LED power and medium MST power. An MST-on time of 15 s was used for analysis (n = 3 independent measurements, error bars represent the standard deviation). b) MST traces recorded for this experiment.

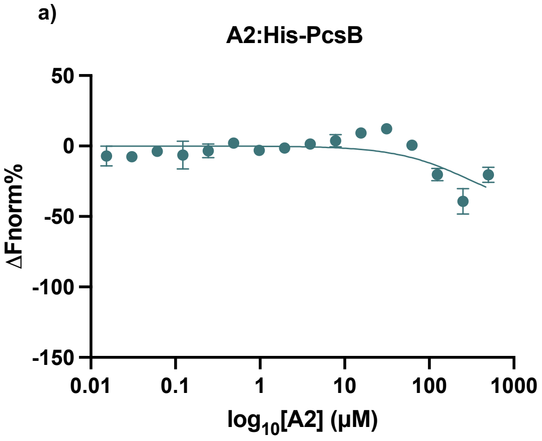


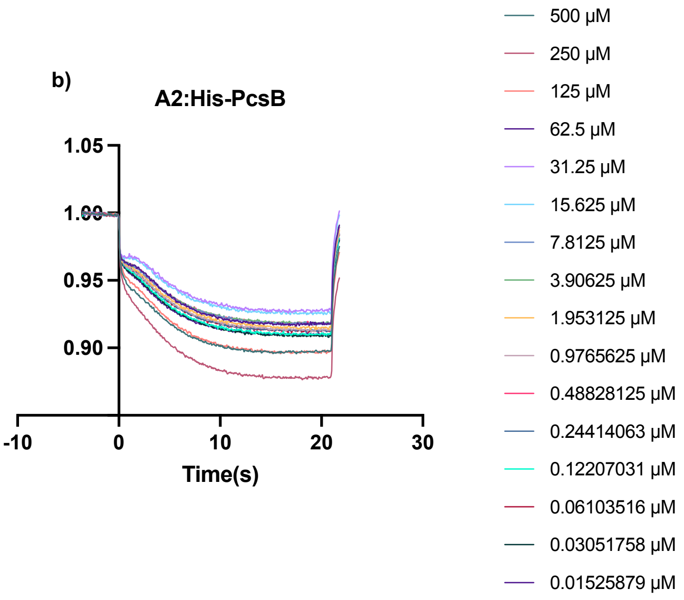


**Figure S19** Microscale thermophoresis of His-PcsB + A2; a) In this MST experiment the concentration of the RED-tris-NTA-labeled His-PcsB was kept constant (10 nM), while the concentration of the non-labeled binding partner (A2) was varied between 0.015 µM – 500 µM or 1000 µM. After 10 min incubation the

samples were loaded into Monolith NT.115 Premium Capillaries (NanoTemper Technologies) and the MST measurement was performed using the Monolith NT.115Pico (NanoTemper Technologies) at 20 % LED power and medium MST power. An MST-on time of 15 s was used for analysis (n = 3 independent measurements, error bars represent the standard deviation). . b) MST traces recorded for this experiment.

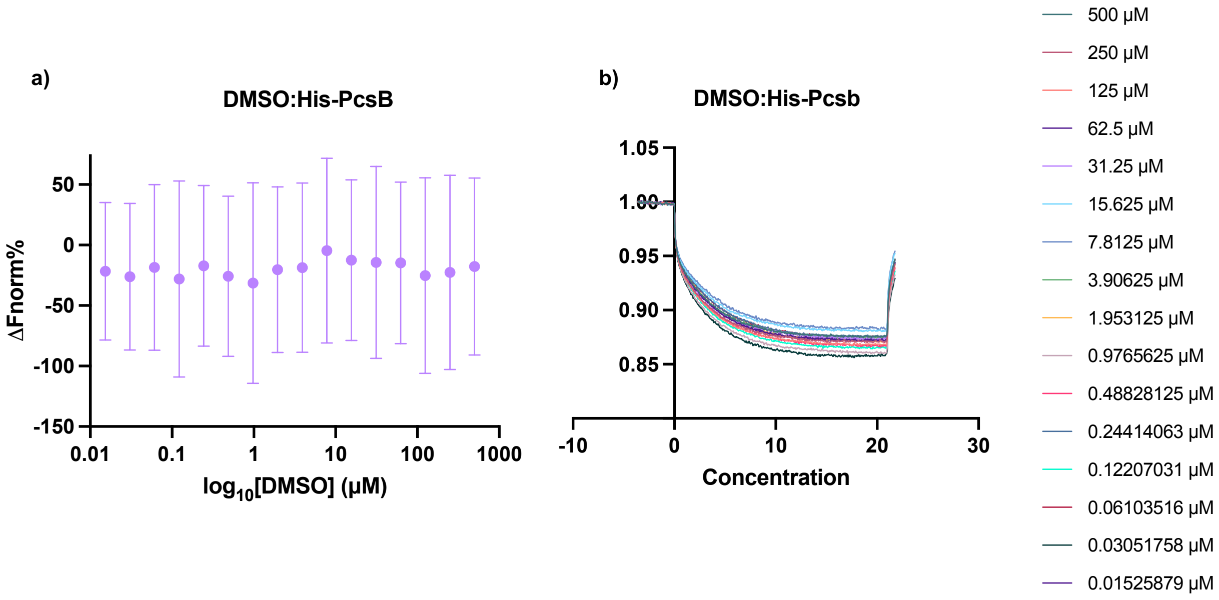


**Figure S20** Microscale thermophoresis of His-PcsB + DMSO; a) In this MST experiment the concentration of the RED-tris-NTA-labeled His-PcsB was kept constant (10 nM), while the concentration of the non-labeled binding partner (DMSO) was varied between 0.015 µM – 500 µM or 1000 µM. After 10 min incubation the samples were loaded into Monolith NT.115 Premium Capillaries (NanoTemper Technologies) and the MST measurement was performed using the Monolith NT.115Pico (NanoTemper Technologies) at 20 % LED power and medium MST power. An MST-on time of 15 s was used for analysis (n = 3 independent measurements, error bars represent the standard deviation). . b) MST traces recorded for this experiment.

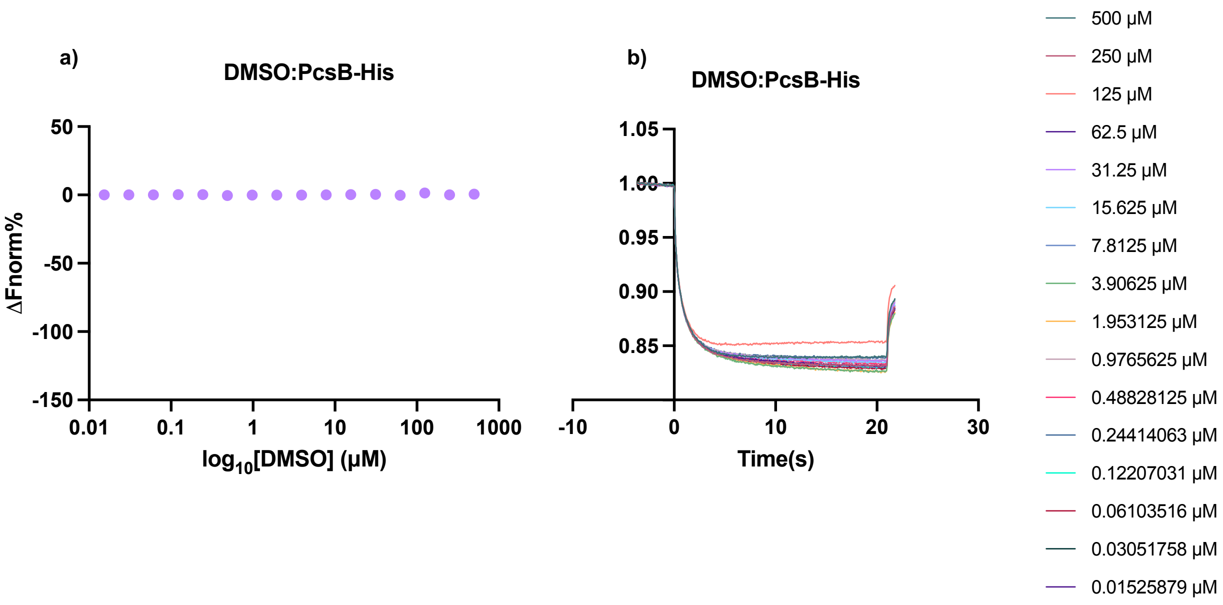


**Figure S21** Microscale thermophoresis of PcsB-His + DMSO.; a) In this MST experiment the concentration of the RED-tris-NTA-labeled PcsB-His was kept constant (10 nM), while the concentration of the non-labeled binding partner (DMSO) was varied between 0.015 µM – 500 µM or 1000 µM. After 10 min incubation the samples were loaded into Monolith NT.115 Premium Capillaries (NanoTemper Technologies) and the MST measurement was performed using the Monolith NT.115Pico (NanoTemper Technologies) at 20 % LED power and medium MST power. An MST-on time of 15 s was used for analysis (n = 3 independent measurements, error bars represent the standard deviation).
Error bars are hidden by the symbol. b) MST traces recorded for this experiment.

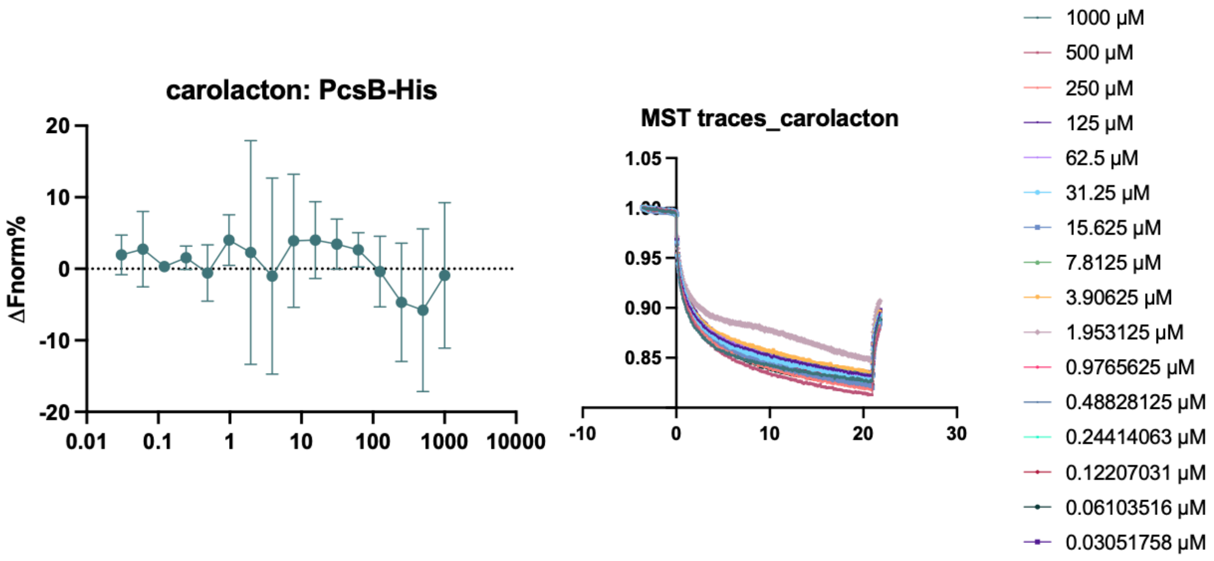


**Figure S22** Microscale thermophoresis of PcsB-His + carolacton; a) In this MST experiment the concentration of the RED-tris-NTA-labeled His-PcsB was kept constant (10 nM), while the concentration of the non-labeled binding partner (DMSO) was varied between 0.015 µM – 500 µM or 1000 µM. After 10

min incubation the samples were loaded into Monolith NT.115 Premium Capillaries (NanoTemper Technologies) and the MST measurement was performed using the Monolith NT.115Pico (NanoTemper Technologies) at 20 % LED power and medium MST power. An MST-on time of 15 s was used for analysis (n = 3 independent measurements, error bars represent the standard deviation). b) MST traces recorded for this experiment.

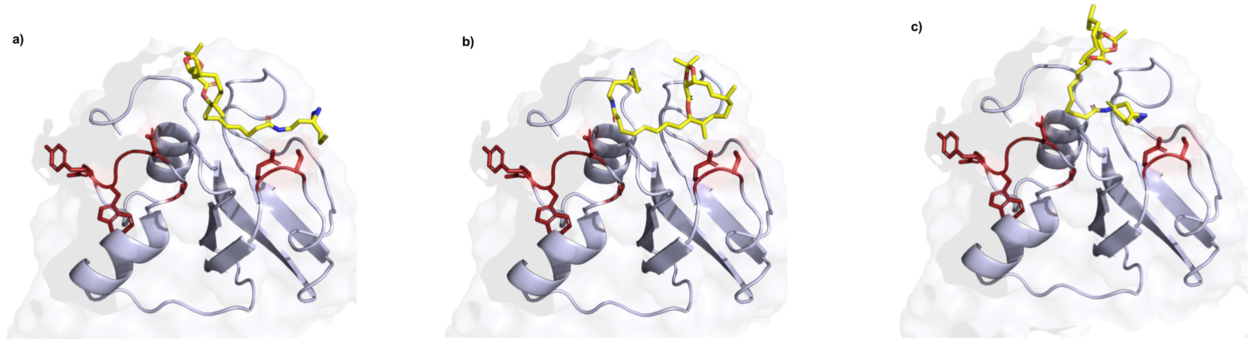


**Figure S23** a-c) Molecular docking of A2PP with the CHAP domain of *spn*PcsB (PDB:4CGK) computed using Glide docking module of Maestro (Schrödinger suite). These three poses were the top scoring poses and highlight the proximity of the diazirine to the crosslinking sites observed experimentally.

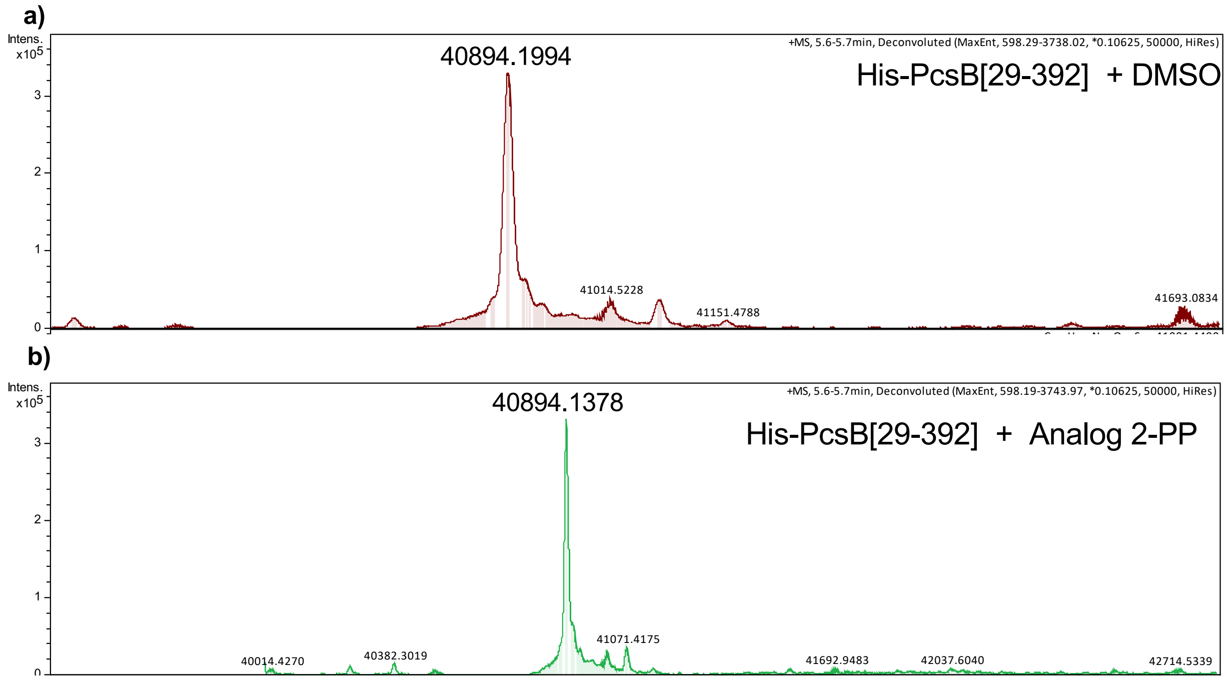


**Figure S24** a) LC-QTOF/MS spectra obtained after 15 min incubation on ice with purified His6-PcsB and DMSO (500 µM), followed by 10 min UV irradiation. b) LC-QTOF/MS spectra obtained after 15 min incubation on ice with purified His6-PcsB and Analog 2-photoprobe (500 µM), followed by 10 min UV irradiation.

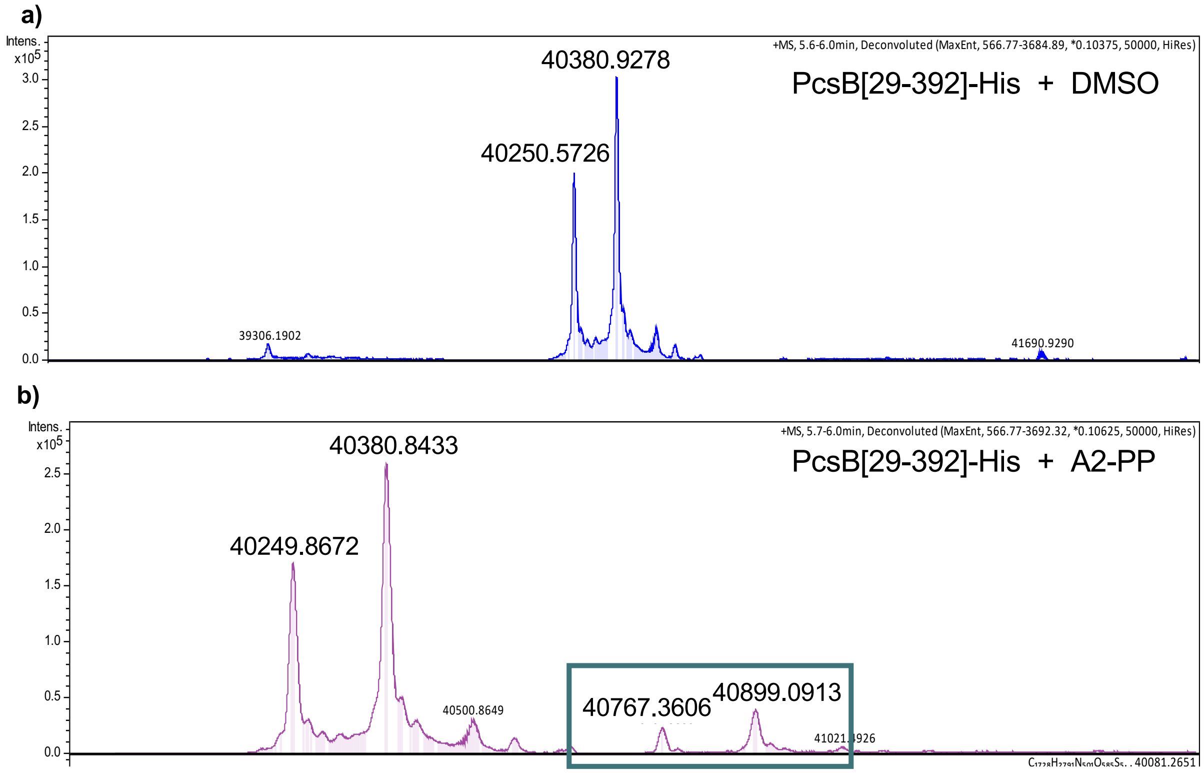


**Figure S25** a) LC-QTOF/MS spectra obtained after 15 min incubation on ice with purified PcsB-His$_6$ and DMSO (500 μM), followed by 10 min UV irradiation. b) LC-QTOF/MS spectra obtained after 15 min incubation on ice with purified His6-PcsB and Analog 2-photoprobe (500 μM), followed by 10 min UV irradiation. Box indicates crosslinked adducts between protein and photoprobe.

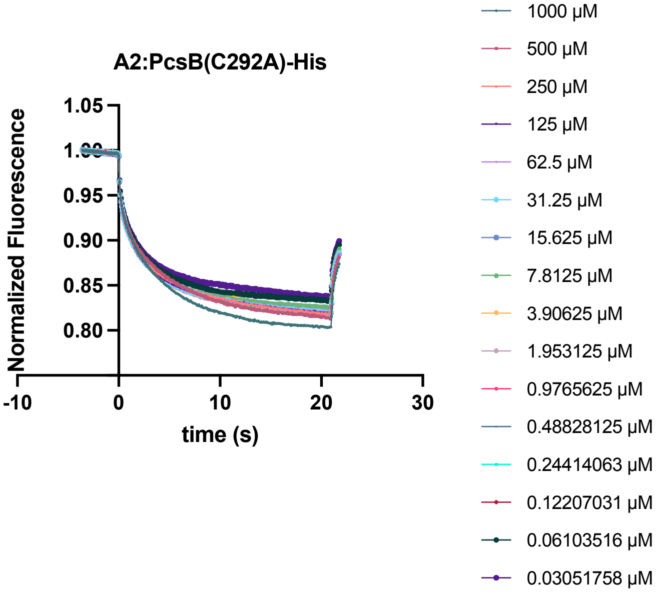

**Figure S26** Microscale thermophoresis traces of PcsB(C292A)-His +A2.; In this MST experiment the concentration of the RED-tris-NTA-labeled PcsB(C292A)-His was kept constant (10 nM), while the concentration of the non-labeled binding partner (A2) was varied between 0.015 µM – 500 µM or 1000 µM. After 10 min incubation the samples were loaded into Monolith NT.115 Premium Capillaries (NanoTemper Technologies) and the MST measurement was performed using the Monolith NT.115Pico (NanoTemper Technologies) at 20 % LED power and medium MST power. An MST-on time of 15 s was used for analysis (n = 3 independent measurements, error bars represent the standard deviation).

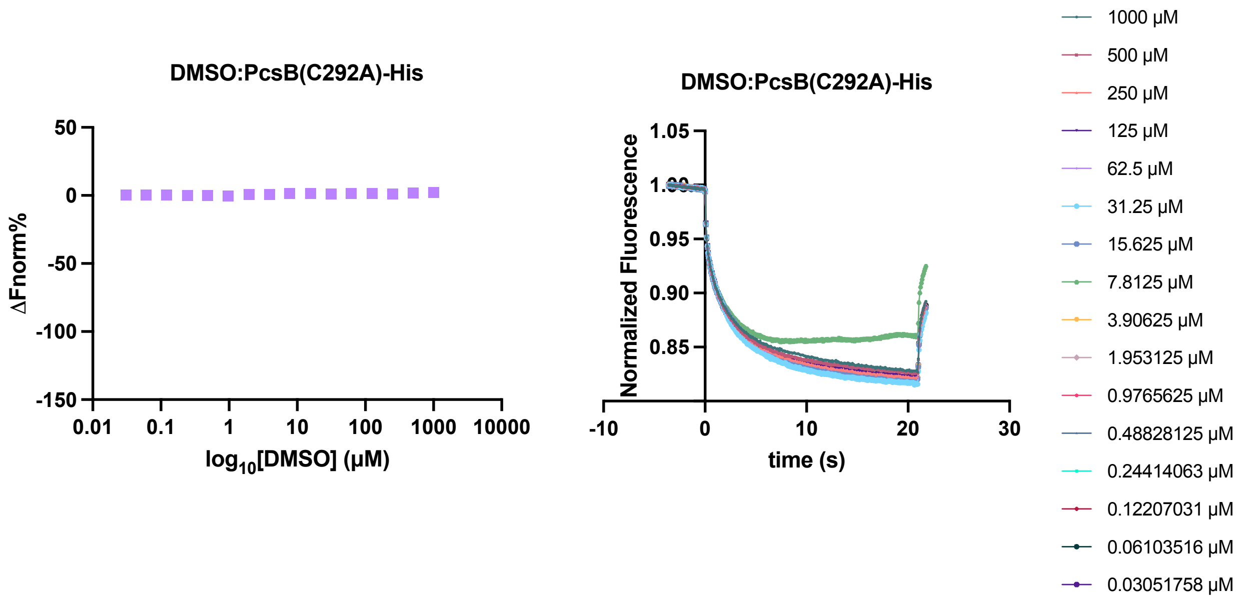


**Figure S27** Microscale thermophoresis of PcsB(C292A)-His + DMSO.; a) In this MST experiment the concentration of the RED-tris-NTA-labeled PcsB(C292A)-His was kept constant (10 nM), while the concentration of the non-labeled binding partner (A2) was varied between 0.015 µM – 500 µM or 1000 µM. After 10 min incubation the samples were loaded into Monolith NT.115 Premium Capillaries (NanoTemper Technologies) and the MST measurement was performed using the Monolith NT.115Pico (NanoTemper Technologies) at 20 % LED power and medium MST power. An MST-on time of 15 s was used for analysis (n = 3 independent measurements, error bars represent the standard deviation). Error bars are hidden by the symbol. b) MST traces recorded for this experiment.

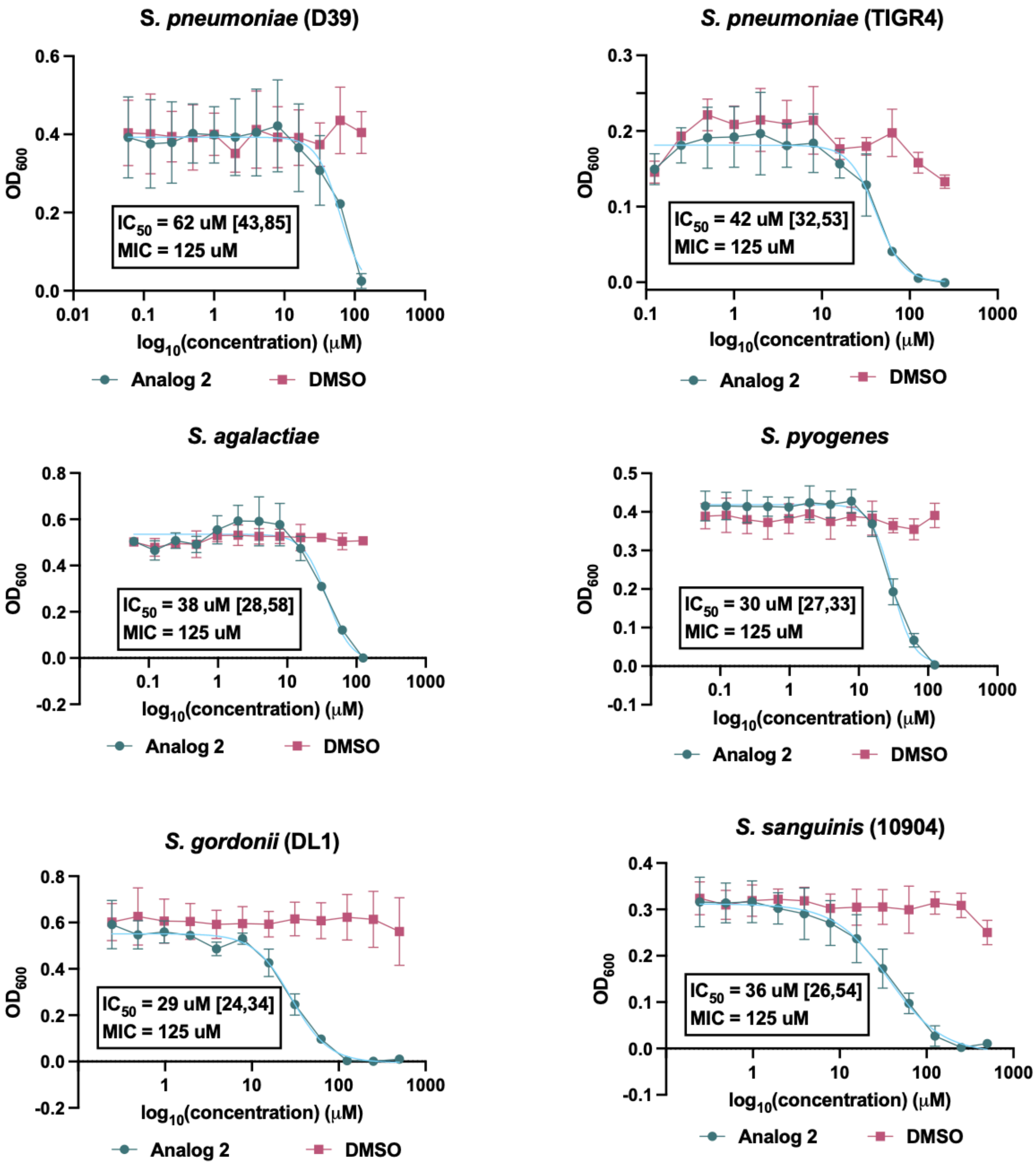


**Figure S28** Minimum inhibitory concentration assay of different *Streptococcus spp.* treated with serially diluted A2 or DMSO. Experiment was conducted in triplicate. All optical density (OD) measurements were performed on a Molecular Devices SpectraMax iD3 plate reader and growth was recorded by $OD_{600}$. $IC_{50}$ curves were calculated with GraphPad Prism using a four-parameter nonlinear regression fit and are represented by the light blue curve. MIC values represent the lowest concentration of A2 that inhibits visible growth. 95% confidence intervals are represented in brackets.

## Chemical Methods

**General notes:** NMR spectra were recorded on an INOVA 500. Chemical shifts are reported in ppm relative to tetramethylsilane and with the indicated solvent as an internal reference. The following abbreviations are used to describe signal multiplicities: s (singlet), d (doublet), t (triplet), q (quartet), m (multiplet), br (broad), dd (doublet of doublets), dt (doublet of triplets), etc. Accurate mass spectra were recorded on a Thermo LTQ FTMS, infrared spectra were obtained using a Thermo Scientific Nicolet iS10 Smart Orbit FT-IR spectrophotometer, and specific rotation measurements were collected using a 1 dm path length using a Perkin Elmer 341 Polarimeter. Non-aqueous reactions were performed under an atmosphere of argon, in flame-dried glassware, with HPLC-grade solvents dried by passage through activated alumina. Brine refers to a saturated aqueous solution of sodium chloride. Reactions monitored via thin-layer chromatography (TLC) using EMD Millipore® TLC silica gel glass plates with $KMnO_4$ stain.

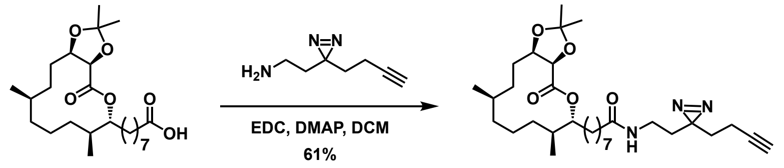


A2 A2-PP

**N-(2-(3-(but-3-yn-1-yl)-3H-diazirin-3-yl)ethyl)-8-((3aR,6R,7S,11R,13aR)-2,2,7,11-tetramethyl -4-oxodecahydro-4H-[1,3]dioxolo[4,5-c][1]oxacyclododecin-6-yl)octanamide (A2-PP).** A flame dried vial was charged with argon, analog 2 (12.17 mg, 0.2853 mmol) and DCM (0.2 mL). The reaction was subsequently cooled to 0°C, then DMAP (0.2 mg, 0.0019 mmol) and EDC (8.0 mg, 0.0380 mmol) were added consecutively. After the reaction stirred for 10 minutes, a solution of the 2-(3-(but-3-yn-1-yl)-3H-diazirin-3-yl)ethan-1-amine, dissolved in DCM (0.2 mL) was added dropwise. The reaction was stirred for 24-30 hours depending on TLC analysis and then added to a separatory funnel containing equal volume of $H_2O$. The organic layer was separated, and the aqueous layer was extracted with DCM (3 x 15 mL). The combined organic layers were washed with brine, dried over $MgSO_4$, filtered and concentrated. Purified by column chromatography (8% EtOAc:DCM + 0.01 % AcOH) and isolated in 61% yield. $R_f$ = 0.76 (20% EtOAc:DCM + 0.01 % AcOH). $^1$H NMR (500 MHz, $CDCl_3$) δ 4.78 (ddd, *J* = 10.1, 6.4, 3.5 Hz, 1H), 4.56 (d, *J* = 6.1 Hz, 1H), 4.37 (ddd, *J* = 9.4, 5.9, 3.1 Hz, 1H), 3.11 (q, *J* = 6.7, 6.2 Hz, 2H), 2.16 (t, *J* = 7.7 Hz, 2H), 2.04 –2.01 (m, 3H), 1.84 (dddd, *J* = 13.8, 10.3, 7.0, 3.3 Hz, 1H), 1.70 (t, *J* = 6.7 Hz, 4H), 1.65 (t, *J* = 7.3 Hz, 4H), 1.63 (s, 3H), 1.62 –1.56 (m, 4H), 1.53 –1.44 (m, 3H), 1.39 (s, 3H), 1.31 –1.20 (m, 13H), 1.19 –1.13 (m, 2H), 1.04 (td, *J* = 10.2, 5.1 Hz, 1H), 0.89 (t, *J* = 7.2 Hz, 6H).$^{13}$C NMR(126 MHz, $CDCl_3$) δ 173.31, 170.13, 109.99, 93.74, 92.66, 82.84, 79.09, 78.16, 77.41, 77.36, 77.16, 76.91, 69.53, 36.86, 36.06, 34.37, 33.64, 32.69, 32.38, 32.32, 29.28, 29.26, 27.98, 27.39, 26.05, 25.73, 24.12, 20.89, 16.95, 13.39. HRMS (ES+): Found 546.39024 (0.19 ppm), $C_{31}H_{51}N_3O_5$ ($M+H^+$) requires 546.39015. IR 3750.37 (N-H), 2927.07 (C-H), 1746.37, 2361.42, 2336.50 (C≡C) 1733.98 (C=O), 1540.05 (N=N) $cm^{-1}$. $[\alpha]^{25}_D$ -16.67 (*c* = 0.18 in $CHCl_3$)

## Biological Methods

**Materials.** *Streptococcus mutans* wild-type strain UA159 was used for all bacterial cultures and was provided by Dr. Bettina Buttaro from Temple University Medical School, Philadelphia, PA. *S. mutans* mutants were provided by Dr. Robert G. Quivey from the Department of Microbiology and Immunology of University of Rochester. Bacterial mutants were maintained on BactoTM Todd-Hewitt agar plates or liquid broth supplemented with erythromycin (5 μg/mL). All optical density (OD) measurements were performed on a Molecular Devices SpectraMax iD3 plate reader.

**Software.** Data were analyzed and visualized using Microsoft Excel (v16.22) and GraphPad Prism (v8.0.1), as well as software specifically listed. NMR data were analyzed using MestReNova (v14.0.1).

DNA and protein sequences were analyzed using SnapGene and Ape. Docking studies were performed with the glide module in Maestro (Schrodinger).

**Folate Dehydrogenase Assay.** *Escherichia coli* FolD (EcFolD) was cloned and expressed as described by Fu et al. Inhibition of the dehydrogenase activity of ecFolD by carolacton, A2 and the A2-PP was investigated using the substrate (6R)-5,10-methylene-5,6,7,8-tetrahydrofolic acid (5,10-CH2-THF) and the cofactor NADP+, while monitoring the formation of 5,10-CH=THF. The dehydrogenase activity was performed as described by Fu et al. with slight modifications.[1] Briefly, (6R,S)-5,10-Methylene-5,6,7,8-tetrahydrofolic acid ((6R,S)-5,10-$CH_2$-THF) calcium salt was used as substrates for FolD dehydrogenase enzyme assay, whereas only the R-isomer (50%) is used by the enzyme. (6R,S)-5,10-$CH_2$-THF was dissolved in $N_2$-sparged buffer (50 mM Tris-HCl (pH 8.0), 100 mM β-mercaptoethanol). The dehydrogenase activity was determined at 30 °C in 50mM Tris-HCl (pH 7.5), 30 mM β-mercaptoethanol (BME) and the concentrations of $NADP^+$ (100 mM stock solution prepared in water) and 5,10-CH2-THF (R-isomer) were fixed at 1 mM and 0.5 mM, respectively. The concentrations of the tested inhibitors varied from 5 nM – 10 µM. The 90 µL reactions of dehydrogenase activity assays were initiated by adding the enzyme to a final concentration of 10 nM and terminated with 100 µL 1M HCl after 2 min incubation at 30 °C. The formation of 5,10-CH=THF was monitored at 350nm (λmax= 350 nm; acidified pH).

**Minimum inhibitory concentration assay for *S. mutans* mutants.** Stock solution of A2 (10 mM), was serial diluted in THB media (pH = 5.5) in flat-bottom 96-well microtiter plates. Each mutant (Quivey, 2015) was grown with compound, and separately with the DMSO vehicle. Mid-exponential phase cell culture was diluted to an $OD_{600}$ of 0.004 and added to the serial diluted compound or DMSO control to reach final volume of 200 µL. Plates were incubated at 37 °C in 5% $CO_2$ for 22 hours (early-stage biofilm) at which time wells were evaluated visually for bacterial growth. $OD_{600}$ measurements of growth was performed after visual inspection. Biological triplicates were performed.

**Biofilm preparation model for *S. mutans* mutants.** Stock solution of compound, 10,000 µM, were serial diluted in THB media with 0.1% sucrose (w/v) in glass flat-bottom 96-well microtiter plates. Each mutant (Quivey, 2015) was grown with compound, and separately with the DMSO vehicle. Mid-exponential phase cell culture was diluted to an $OD_{600}$ of 0.004 and added to the serial diluted compound to reach final volume of 200 µL. Plates were incubated at 37 °C in 5% $CO_2$ for 22 hours (early-stage biofilm) at which time wells were evaluated visually for bacterial growth. $OD_{600}$ measurements of growth was performed after visual inspection. Biological triplicates were performed.

**Confocal Imaging.** Biofilms were prepared with above procedure. To perform efficient imaging, uncoated 96-Well Plates with 5 mm Glass Diameter from MatTek (Part No: P96G-0-5-F) were used for confocal imaging experiments. After incubation, media was removed, and each well was carefully rinsed three times with PBS to remove planktonic cells. Subsequently, 20 µL of BacLight LIVE/DEAD™ stain was added to each well. Excess dye was rinsed off biofilm with PBS. Images of biofilms were then obtained using the Olympus FV1000 inverted microscope in the Integrated Cellular Imaging Core at Emory University.

**Label-free AfBPP in growing biofilm.** 5 mL of THB media was inoculated with *S. mutans* (UA159) from freezer stock and grown overnight. The overnight culture was diluted (1:100) and regrown to $OD_{600}$ = 0.4 (exponential phase). To a petri dish, 200 µl of 100X stock solution of A2-PP was added to 19.8 mL of THB sucrose 0.1% sucrose (w/v). Petri dishes were incubated at 37 °C in 5% $CO_2$ for 24 hours. Petri dishes were then immediately irradiated with UV light (280-315 nm). Irradiation cycle was repeated three times (6 minutes irradiation, 6 minutes on cold pack). Then the biofilm supernatant was removed, and the biofilm was carefully rinsed with PBS three times. Biofilm cells were resuspended with 10 mL of PBS and transferred to 25 mL falcon tubes. Samples were centrifuged for 10 minutes (6000 x g, 4°C), supernatant discarded, and pellet was transferred to Eppendorf tube with 800 µL of PBS. The samples were then again centrifuged for 10 minutes (6,000 g, 4°C), supernatant was disposed, and pellet was resuspended in PBS

with 0.4% SDS (4 °C). Bacterial cell lysis was completed with 3 cycles of 30 second sonification at 80% intensity. Samples were then centrifuged at 20,000 x g for 30 minutes (4 °C) thus separating the soluble fraction (supernatant) and the insoluble fraction (pellet). Samples were then processed according to the gel-based AfBPP or the gel-free AfBPP procedure described below.

**Gel-based AfBPP.** With the soluble fraction, we performed click chemistry with rhodium azide using a freshly prepared "master mix". Master mix included (per sample) 2 µl of $RhN_3$ (Tetramethylrhodamine (TAMRA Azide (Tetramethylrhodamine 5-Carboxamido-(6-Azidohexanyl) (10 mM in DMSO), 2 µl TCEP (52 mM, 15 mg/mL in dd $H_2O$), and 6 µl TBTA ligand (1.677 M 1 x ligand; 800 uL t-BuOH, 180 µL DMSO, 20 µL 50 x ligand) (50 x ligand = 8.85 mg in 200 uL DMSO). 88 µL of the soluble fraction and 10 µL of the master mix were combined in an Eppendorf and vortexed. Then, 2 µL of 50 mM CuSO4 were added to each sample and vortexed. Samples were incubated for 1 hour at RT in the dark. 100 µl of 2 X SDS loading buffer were added to the samples, vortexed analyzed by SDS-PAGE (12.5%) with fluorescence scanning. Protein loading was visualized by Coomassie-staining of the gels.

**Gel-free and label-free AfBPP.** Protein concentration was measured with the BCA Assay and samples were adjusted to 0.63 mg/mL using PBS + 0.4% SDS buffer (total volume of 500 µL of each sample). Click chemistry was performed with biotin azide using a freshly prepared "master mix". Master mix included (per 500 µL sample) 3 µL of biotin azide (10 mM in DMSO), 10 µL TCEP (52 mM, 15 mg/mL in dd H2O), and 30 µL TBTA ligand (1.677 M 1 x ligand; 800 uL t-BuOH, 180 µl DMSO, 20 µL 50 x ligand) (50 x ligand = 8.85 mg in 200 uL DMSO), and 10 µl of $CuSO_4$ (50 mM stock in dd $H_2O$). 500 µL of the soluble fraction and 53 µL of the master mix were combined in a 15 mL falcon tube, vortexed and incubated for 1 hour at RT in the dark. Proteins were precipitated by adding 4x volume (2 mL) cold acetone (-80 °C). Samples were then stored at -20 °C overnight). Precipitated proteins were pelletized for 15 minutes at 16900 x g at 4 °C and the supernatants were discarded. Protein pellets were washed twice with 500 µL of cold methanol (-80 °C) and resuspension with sonication (10 seconds, 10% intensity, Sonopuls HD 2070 ultrasonic rod, Bandelin electronic GmbH). Pelletize protein for 15 minutes at 16900 x g at 4 °C, discard supernatant and resuspend protein pellet in 500 µL 0.4% SDS in PBS (at RT) by sonication (10 seconds, 10% intensity, Sonopuls HD 2070 ultrasonic rod, Bandelin electronic GmbH). Protein enrichment was then started by transferring 50 µL of Avidin bead suspension into Protein LoBind Eppendorf tubes with a cropped pipette tip. Beads were washed with 1 mL of 0.4% SDS (MS grade) in PBS three times (3 minutes, 400 x g). Incompletely solubilized protein aggregates were removed with centrifugation, and then 0.5 mL of protein sample was transferred to the LoBind Eppendorf tubes containing the Avidin beads. Samples were incubated at RT with continuous mixing for 1 hour. To remove any bound proteins, beads were then washed three times with 1 mL 0.4% SDS in PBS (MS grade), two times with 1 mL 6 M urea in dd$H_2O$ (MS grade) and three times with 1 mL PBS (MS grade). For quantitative mass spectrometric analyses, avidin agarose beads with bound proteins were resuspended in200 uL of X buffer (7 M urea, 2 M thiourea in 20 mM HEPES buffer pH 7.5). Upon on-bead reduction with TCEP (5 mM) for 1 hour at 37 °C, proteins were alkylated using of iodoacetamide (10 mM) at RT for 30 minutes in the dark and samples were quenched with dithiothreitol (DTT, 10mM at RT for 30 min. The protein samples were digested with LysC (1 µL per sample) for 2-4 hours at RT. Samples were then diluted with 600 µL of 50 mM of TEAB, 1.5 µL of 0.5 µg/µL trypsin (sequencing grade, modified, *Promega*) was added and the samples were incubated overnight at 37 °C under continuous shaking (450 rpm). On the next day, digestion was stopped by adding 10 µL of FA to the samples. The pH was checked (3 or below). Samples were centrifuged to pelletize the beads. Samples were desalted by using 50 mg SepPak C18 columns (Waters) equilibrated with equilibrated with 1 mL of 0.1% TFA. Samples were loaded and the peptides were washed three times with 1 mL 0.1% TFA, and then 500 µL 0.5% FA. Finally, the peptide samples were then eluted off the column with 250 µL 80% MeCN/0.5% FA, three times, lyophilized and stored at -80 °C until further usage.

**LC-MS/MS analysis.** Before MS measurements, the lyophilized peptides were resolved in 25-40 µL 1% FA and filtered through 0.22 µm PVDF filters (*Millipore*), which were equilibrated with 300 µL 1% FA. The filtrates were transferred into MS-vials and stored at –20 °C until the measurements were performed.

Samples were analyzed with an UltiMate 3000 nano HPLC system (*Dionex*) using an Acclaim C18 PepMap100 (75 µm ID × 2 cm) trap column and an Acclaim PepMap RSLC C18 (75 µm ID × 50 cm) separation column coupled to a Q Exactive Plus (*Thermo Fisher*) in EASY-spray setting. Samples were loaded on the trap column and washed with 0.1% TFA, then transferred to the analytical column (buffer A: $H_2O$ with 0.1% FA, buffer B: ACN with 0.1% FA, flow 300 nL/min, gradient 5 to 22% buffer B in 115 min, then to 32% buffer B in 10 min, then to 90% buffer B in 10 min and hold 90% buffer B for 10 min, then to 5% buffer B in 0.1 min and hold 5% buffer B for 9.9 min). Q Exactive Plus was operated in a TOP10 data dependent mode. Full scan acquisition was performed in the orbitrap at a resolution of 140,000 and an AGC target of $3e^6$ (maximum injection time of 80 ms) in a scan range of 300-1,500 m/z. Monoisotopic precursor selection as well as dynamic exclusion (exclusion duration: 60 s) was enabled. Precursors with charge states of >1 and intensities greater than $1e^5$ were selected for fragmentation. Isolation was performed in the quadrupole using a window of 1.6 m/z. Precursors were collected to an AGC target of $1e^5$ (maximum injection time of 100 ms) and acquisition was performed at a resolution of 17,500 in a scan range of 200-2,000 m/z. Fragments were generated using higher-energy collisional dissociation (HCD, normalized collision energy: 27%) and detected in the orbitrap.

**MS-data analysis.** Raw files were analyzed using MaxQuant software (version 1.6.2.10) with the Andromeda search engine. The following settings were applied: fixed modification: carbamidomethylation (cysteine); variable modification: oxidation (methionine), acetylation (*N*-terminus); proteolytic enzyme: trypsin/P; missed cleavages: 2; main search tolerance: 4.5 ppm; MS/MS tolerance: 0.5 Da; false discovery rates: 0.01. The options "LFQ" and "match between runs" (0.7 min match and 20 min alignment time windows) were enabled; "second peptides" was disabled (Cox, 2014) . Searches were performed against the UniProt database for *S. mutants* UA159 (taxid: 210007, 29th July 2019).

Statistical analysis of the data was performed using Perseus (version 1.6.14.0.) (Tyanova, 2016). Putative contaminants, reverse peptides and peptides only identified by site were deleted. LFQ intensities were $log_2$-transformed, and data was filtered for three valid values in at least one group and missing value imputation was performed over the total matrix. For statistical evaluation, $-log_{10}$($P$ values) were obtained by a two-sided two sample Student's $t$-test.

**Construction of overexpression strains in *S. mutans* UA159.** The full length of GbpB gene was amplified by PCR using primer set GbpB-Sal1-F (TCAGTCGACATGAAAAAAAGAATTTTATCA) and GbpB-Kpn1-R (TCAGGTACCTTAGTTTGGATAGATATAGCT). The amplified PCR fragments were digested with *Sal*I and *Kpn*I, and then ligated into *E. coli*-Streptococcal shuttle vector pVPT (erythromycin resistant) (Zhou, Fives-Taylor et al. 2008), yielding pVPT-GbpB. The resulting plasmid was selected by LB plate with erythromycin resistance and confirmed by sequencing. The pVPT-GbpB was transformed into *S. mutans* UA159 for GbpB overexpression studies.

**Biofilm formation assay for overexpression.** *S. mutans* biofilm was grown in Brain Heart Infusion (BHI). Overnight cultures were subcultured into fresh BHI, grown to an optical density at 600 nm ($OD_{600}$) of 0.6. It subsequently diluted at 1:100 to 200uL biofilm medium in a polystyrene 96-well plate (Nunc, Thermo Scientific), and grown at 5% $CO_2$ at 37°C under static conditions The $OD_{600}$ was measured to evaluate growth effects. Biofilm samples were then collected after 16 h and stained with 0.1% crystal violet for 15 min. The well was rinsed 3 times and the crystal violet was solubilized in 200 µL of 30% acetic acid. The $OD_{562}$ was used to measure biofilm formation (Wu, Zeng et al. 2007).

**Protein expression and purification.** Plasmids were confirmed using Sanger sequencing and transformed into BL-21(DE3). *E. coli* BL21 (DE3) containing the desired expression plasmid was inoculated into LB-Miller broth with the appropriate antibiotic and grown at 37 °C overnight. The overnight culture was diluted 200-fold into LB-Miller broth containing antibiotic, $MgSO_4$ and glycerol. The cells were grown at 37 °C until $OD_{600}$ reached 0.6, and isopropyl-1thio-β-D-galactopyranoside (IPTG) was

added to a final concentration of 100 μM and incubated for 4 hours at 30 ºC or overnight at 16 ºC. Following incubation, cells were harvested by centrifugation at 5020 x g for 30 minutes. Cell pellets were washed with 150 mL 20 mM Tris pH 8 containing 100 mM NaCl and flash-frozen with liquid nitrogen, and then thawed. Pellets were resuspended in 1mM PMSF, 1 tablet of Roche cOmplete protease inhibitor cocktail, 1:100 lysosyme (100 mg/mL) and 1:100 DNAse (50 mg/mL). Cells were homogenized using an Ultraturrax and lysed by three passages through an Avestin EmulsiFlex-C5 disruptor. Cell lysates were clarified by centrifugation at 47,000 x g for 60 minutes. The supernatants were applied to preequilibrated nickel-NTA resin. Protein was eluted with 3 column volumes of 20 mM Tris pH 8 containing 100 mM NaCl. Proteins were concentrated using 30 molecular weight cut-off (MWCO) Amicon Ultra centrifuge filter device (Millipore). The supernatant was purified by size-exclusion chromatography using an ÄKTA-pure (Cytiva) and a Superdex 200 increase column in 20 mM Tris pH 8 containing 100 mM NaCl. The desired protein fractions were collected and concentration to 1 mg/mL. Purified protein concentrations were evaluated by NanoDrop 2000 (ThermoFisher) using the Protein A280 method. Following purification by affinity column and SEC, protein fractions were assayed for purity by separation on 4-20 % SDS-PAGE (sodium dodecyl sulfate - polyacrylamide gel electrophoresis) and either Coomassie staining or Western blot. For Western blotting, the gel was transferred to methanol-activated polyvinylidene fluoride (PVDF) membranes (Bio-Rad Laboratories). The membrane was blocked with 10 mL casein blocking buffer and probed with α-His-HRP (1:5,000, BioLegend). Proteins were detected using the Amersham enhanced chemiluminescence (ECL) reagent and imaged on an Azure c400.

**Microscale Thermophoresis (MST).** All $His_6$-tagged proteins were labeled using the Protein Labeling Kit RED-NHS (NanoTemper Technologies). The labeling reaction was performed according to the manufacturer's instructions. The labeled proteins were adjusted to 10 nM with 50 mM HEPES buffer (150 mM NaCl, pH = 7.4) supplemented with 0.05 % Tween 20 (unless otherwise noted). Ligands were prepared as a DMSO stock from either a 10 mM stock or a 50 mM stock and diluted in 50 mM HEPES buffer (150 mM NaCl, pH = 7.4) supplemented with 0.05 % Tween 20, and a series of 16 1:1 dilutions was prepared using the same buffer, producing ligand concentrations ranging from 0.03 μM to 1000 μM. For the measurement, each ligand dilution was mixed with one volume of labeled protein, which led to a final concentration of protein of 10 nM and final ligand concentrations ranging from 0.015 μM to 500 μM. After 10 min incubation followed by centrifugation at 15 000 × g for 10 min at 4 °C, the samples were loaded into Monolith NT.115 [Premium] Capillaries (NanoTemper Technologies). The MST measurements were performed using a Monolith NT.115 Pico instrument (NanoTemper Technologies) at an ambient temperature of 25 °C. Instrument parameters were adjusted to 20 % LED power and medium MST power. An MST-on time of 15 s was used for analysis. To determine dissociation constants, we employed the $K_d$ binding model included in the MO.Affinity Analysis Software provided by Nanotemper Technologies.

**MST Data Analysis.** Data of three (unless otherwise noted) independently pipetted measurements were analyzed (MO.Affinity Analysis software version 2.3, NanoTemper Technologies) using the signal from an MST on time of 1.5 s. We used a conservative definition of outliers, only removing data points where there were irregularities with the absolute fluorescence, capillary scan showed irregularities, MST or temperature related intensity change (TRIC) traces showed bleaching, or aggregation. The dissociation constants (Kd) were determined using the $K_d$ binding model (Eq. 1), which is based on the Langmuir binding isotherm and is defined as follows: f(c) is the fraction bound at given ligand concentration c. Unbound is defined as the normalized fluorescence signal (Fnorm; MST mode) of the target alone, and bound is the normalized fluorescence of the ligand:protein complex. Kd is the dissociation constant or binding affinity, and c(target) is final concentration of the target present in the assay. The concentration used of the target was 10 nM.

$$f(c) = Unbound + \frac{(Bound - Unbound) * c + c(target) + Kd - \sqrt{(c + c(target) + Kd)^2 - 4c * c(target)}}{2c(target)} \quad \text{Eq. (1)}$$

Data was processed for presentation using baseline corrected normalized fluorescence $\Delta F_{norm}$ [%], wherein the baseline $F_{norm}$ value is subtracted from all data points of the same curve. This value is

provided by MO.Affinity Analysis as the 'unbound' value when a fit is performed. In cases where a fit was not successful, the average of capillaries 14-16 was taken and used as the baseline $F_{norm}$ value.

**In vitro photocrosslinking.** In a 96 well plate, compound (10 μM) or DMSO was added to 100 mL of either His-PcsB or PcsB-His (1 mg/mL) in 20 mM Tris buffer to give a final concentration of 500 mM to ensure saturation. Samples were incubated for 15 minutes on ice. Photo-crosslinking was performed by using a UVP Blak-Ray B-100AP high-intensity UV lamp with a 100-W spot bulb. The lamp was positioned approximately 6 cm away from the samples and the samples were irradiated for 10 minutes. Samples were then split and transferred to 1.5 mL Eppendorf tubes. Subsequently, samples were either analyzed directly by ESI-qTOF-MS for intact protein analysis or further processed for trypsin digest analysis. Molecular weight determination of intact protein was conducted by direct Electro Spray Ionization (ESI) on the Q-TOF Premier and analyzed by the Harvard Center for Mass Spectrometry Proteomics Center.

**Processing for proteolytic digest.** To prepare samples for trypsin digest, a methanol-chloroform precipitation procedure was employed. Methanol (50 μL) was added to the sample (50 μL) and vortexed. Chloroform (50 μL) was added, and the sample vortexed. The sample was centrifuged for 2 min at 14000 g, and the top layer aspirated off. Methanol (200 μL) was added, the sample was vortexed, centrifuged for 3 minutes at 14000g, and submitted and analyzed by LCMS/MS by the Harvard Center for Mass Spectrometry Proteomics Center.

**Molecular docking.** Docking was conducted using the Glide grid-based docking module of Maestro (Schrödinger suite) in extra-precision (XP) mode. The refined structure of PcsB downloaded from the PDB database was optimized in Maestro using the Protein Preparation Wizard (Schrodinger, 2010). Ligands were prepared using the LigPrep module of the Schrodinger suite using the OPLS4 force field. The receptor grid was generated by defining the binding site using the cocrystalized ligand. An outer box of 40 X 40 X 40 Å and an inner box of 10 X 10 X 10 Å was used.

**Minimum inhibitory concentration assay for Streptococci spp.** Stock solution of A2 (10 mM), was serial diluted in Todd-Hewitt Broth supplemented with 5% yeast extract in flat-bottom 96-well microtiter plates. Each strain was grown with compound, and separately with the DMSO vehicle. Mid-exponential phase cell culture was diluted to an $OD_{600}$ of 0.004 and added to the serial diluted compound or DMSO control to reach final volume of 200 μL. Plates were incubated at 37 °C in 5% $CO_2$ for 22 hours (early-stage biofilm) at which time wells were evaluated visually for bacterial growth. $OD_{600}$ measurements of growth was performed after visual inspection. Biological triplicates were performed.

## References


Cox J, Hein MY, Luber CA, Paron I, Nagaraj N, Mann M. Accurate proteome-wide label-free quantification by delayed normalization and maximal peptide ratio extraction, termed MaxLFQ. *Mol Cell Proteomics.* **2014**,*13*(9), 2513-26. DOI: 10.1074/mcp.M113.031591.

Fu, C., Sikandar, A., Donner, J. *et al.* The natural product carolacton inhibits folate-dependent C1 metabolism by targeting FolD/MTHFD. *Nat Commun.* **2017**, *8*, 1529. DOI: 10.1038/s41467-017-01671-5

Quivey RG Jr, Grayhack EJ, Faustoferri RC, Hubbard CJ, Baldeck JD, Wolf AS, MacGilvray ME, Rosalen PL, Scott-Anne K, Santiago B, Gopal S, Payne J, Marquis RE. Functional profiling in Streptococcus mutans: construction and examination of a genomic collection of gene deletion mutants. *Mol Oral Microbiol.* **2015**, *30* (6), 474-495. doi: 10.1111/omi.12107.

Tyanova, S., Temu, T., Sinitcyn, P. *et al.* The Perseus computational platform for comprehensive analysis of (prote)omics data. *Nat Methods.* **2016**, *13*, 731–740. DOI: 10.1038/nmeth.3901

Wu H, Zeng M, Fives-Taylor P. The glycan moieties and the N-terminal polypeptide backbone of a fimbria-associated adhesin, Fap1, play distinct roles in the biofilm development of Streptococcus parasanguinis. *Infect Immun.* **2007,** *75*(5), 2181-8. DOI: 10.1128/IAI.01544-06.

Zhou M, Fives-Taylor P, Wu H. The utility of affinity-tags for detection of a streptococcal protein from a variety of streptococcal species. *J. Microbiol. Methods.* **2008**, *72*(3), 249-56. DOI: 10.1016/j.mimet.2007.12.00

## Compound spectra

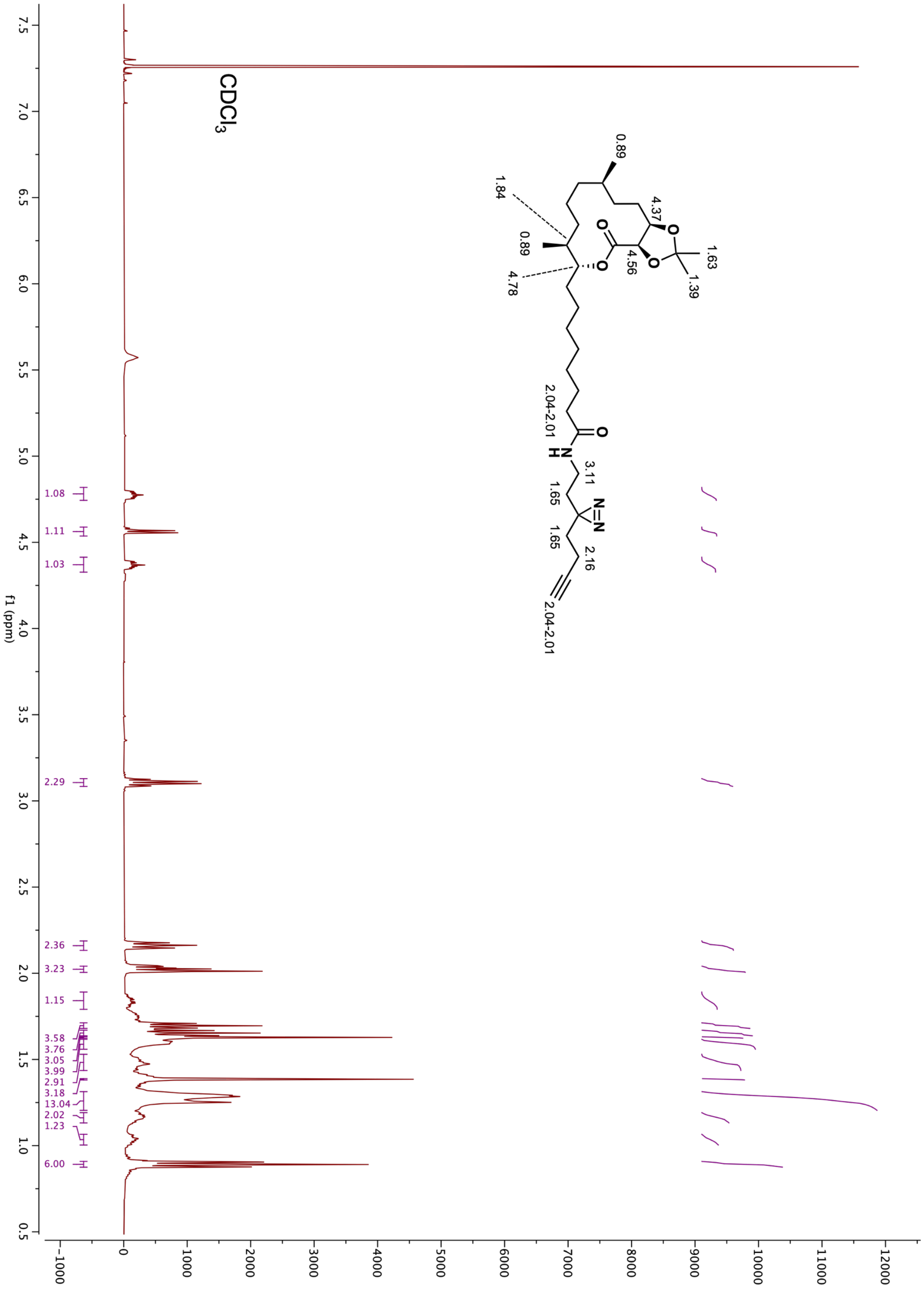
CDCl3
f1 (ppm)
0.89
1.84
4.37
4.56
0.89
4.78
1.63
1.39
2.04-2.01
3.11
1.65
1.65
2.16
2.04-2.01
1.08
1.11
1.03
2.29
2.36
3.23
1.15
3.58
3.76
3.05
3.99
2.91
3.18
13.04
2.02
1.23
6.00

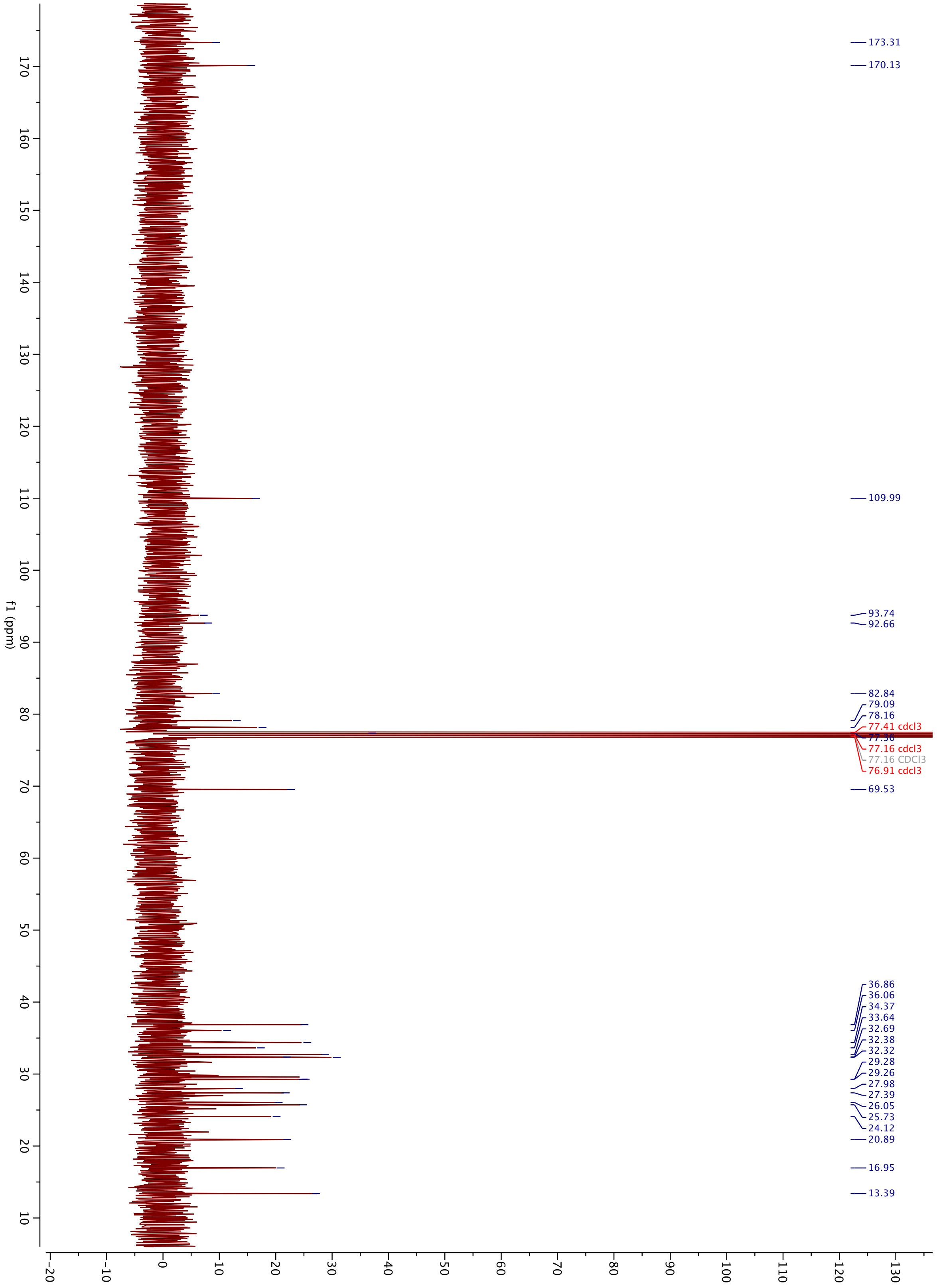
173.31
170.13
109.99
93.74
92.66
82.84
79.09
78.16
77.41 cdcl3
77.36
77.16 cdcl3
77.16 CDCl3
76.91 cdcl3
69.53
36.86
36.06
34.37
33.64
32.69
32.38
32.32
29.28
29.26
27.98
27.39
26.05
25.73
24.12
20.89
16.95
13.39
f1 (ppm)

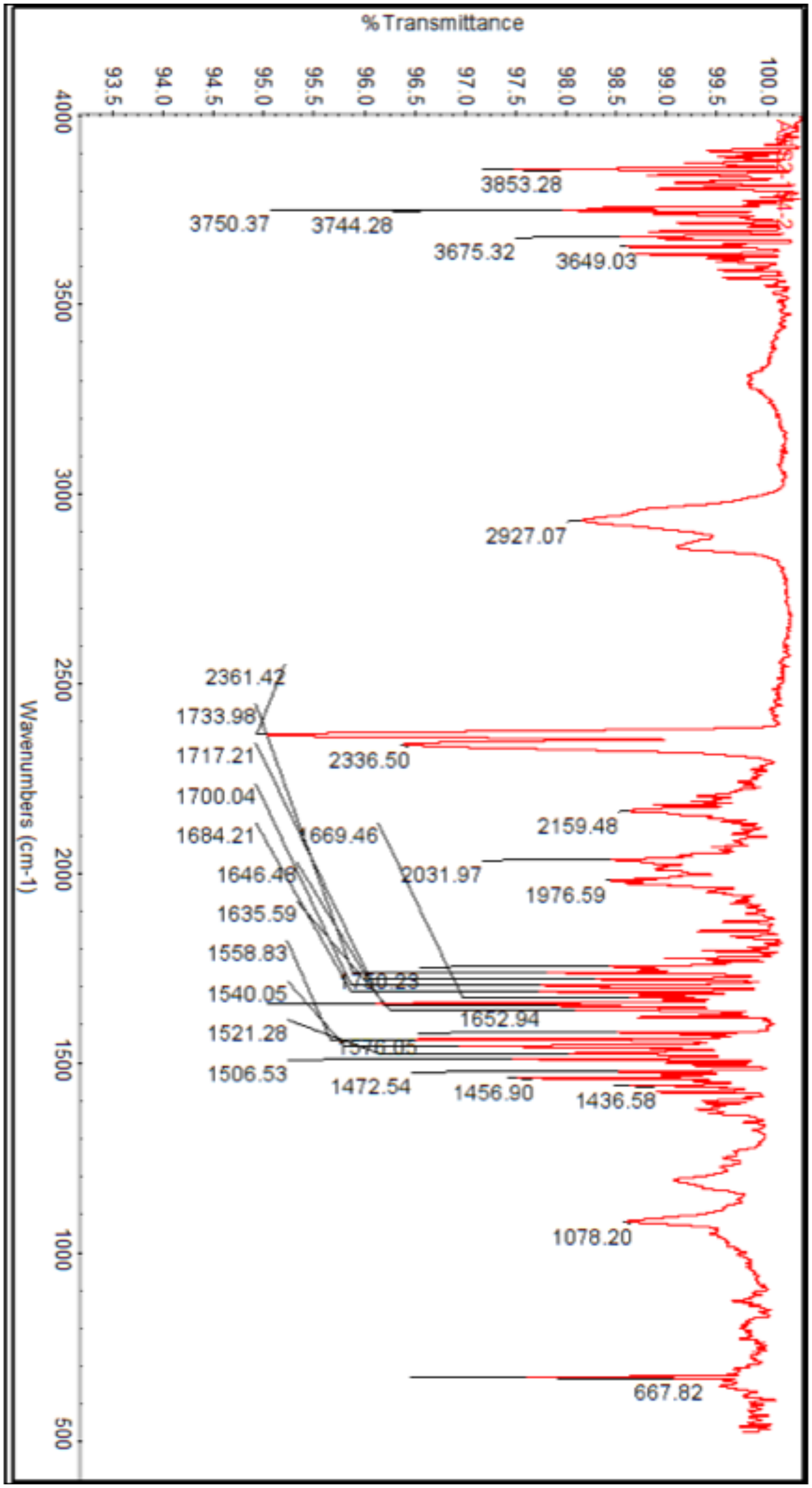

% Transmittance
Wavenumbers (cm-1)
3853.28
3750.37
3744.28
3675.32
3649.03
2927.07
2361.42
1733.98
2336.50
1717.21
1700.04
2159.48
1684.21
1669.46
2031.97
1646.46
1976.59
1635.59
1558.83
1750.23
1540.05
1652.94
1521.28
1576.05
1506.53
1472.54
1456.90
1436.58
1078.20
667.82
